# Novel subgroups of obesity and their association with outcomes: a data-driven cluster analysis

**DOI:** 10.1186/s12889-024-17648-1

**Published:** 2024-01-09

**Authors:** Saki Takeshita, Yuichi Nishioka, Yuko Tamaki, Fumika Kamitani, Takako Mohri, Hiroki Nakajima, Yukako Kurematsu, Sadanori Okada, Tomoya Myojin, Tatsuya Noda, Tomoaki Imamura, Yutaka Takahashi

**Affiliations:** 1 Department of Public Health, Health Management and Policy, Nara Medical University, 840 Shijo-Cho, Kashihara Nara, 634-8521 Japan; 2 Department of Diabetes and Endocrinology, Nara Medical University, 840 Shijo-Cho, Kashihara, Nara 634-8521 Japan

**Keywords:** Obesity, Diabetes, Cluster analysis, Heterogeneity, Prognosis

## Abstract

**Background:**

Obesity is associated with various complications and decreased life expectancy, and substantial heterogeneity in complications and outcomes has been observed. However, the subgroups of obesity have not yet been clearly defined. This study aimed to identify the subgroups of obesity especially those for target of interventions by cluster analysis.

**Methods:**

In this study, an unsupervised, data-driven cluster analysis of 9,494 individuals with obesity (body mass index ≥ 35 kg/m^2^) was performed using the data of ICD-10, drug, and medical procedure from the healthcare claims database. The prevalence and clinical characteristics of the complications such as diabetes in each cluster were evaluated using the prescription records. Additionally, renal and life prognoses were compared among the clusters.

**Results:**

We identified seven clusters characterised by different combinations of complications and several complications were observed exclusively in each cluster. Notably, the poorest prognosis was observed in individuals who rarely visited a hospital after being diagnosed with obesity, followed by those with cardiovascular complications and diabetes.

**Conclusions:**

In this study, we identified seven subgroups of individuals with obesity using population-based data-driven cluster analysis. We clearly demonstrated important target subgroups for intervention as well as a metabolically healthy obesity group.

**Supplementary Information:**

The online version contains supplementary material available at 10.1186/s12889-024-17648-1.

## Background

The World Health Organisation (WHO) reported an estimate of > 650 million individuals with obesity in 2016 and that the obesity rate has closely tripled worldwide since 1975. Approximately 13% of the world’s adult population (11% of men and 15% of women) had obesity in 2016 [1]. Notably, obesity has been reported to have an almost similar association with chronic health conditions as does twenty years [2]. Obesity is associated with diverse complications [3] and various chronic diseases, such as type 2 diabetes, hypertension, cardiovascular diseases, dyslipidaemia, non-alcoholic fatty liver disease, chronic kidney disease, and obstructive sleep apnoea [4]. Obesity has also been reported to be associated with an increased risk of end-stage renal disease, which requires lifelong dialysis [5–8]. Owing to the global disease burden, more than 4 million people die yearly due to obesity, with increased mortality [9]. The impact of obesity on health may differ according to race. Therefore, ethnicity-specific body mass index (BMI) cut offs should be set to provide optimal intervention and management for obesity [10]. In Japan, individuals with a BMI of ≥ 35 kg/m^2^ are defined as ‘severely obese’ with significant complications and qualified for bariatric surgery [11, 12]. In addition, the limited accuracy of BMI as an obesity biomarker has been indicated in the previous studies [13, 14].

Furthermore, obesity greatly impacts healthcare costs, resulting in a socioeconomic burden. For example, the increased prevalence of obesity accounts for the increased medical spending of approximately $40 billion between 1998 and 2006 [15]. Furthermore, obesity is associated with a 36% increase in inpatient and outpatient spending and a 77% increase in medication costs [2]; therefore, appropriate and efficient intervention, including patient selection, is needed.

The clinical heterogeneity of obesity has recently been described [16]. Individuals with cerebrovascular complications, such as myocardial infarction and stroke, generally exhibit increased mortality risk [17], and visceral adiposity has been suggested to be involved in at least a part of these pathological conditions as an underlying mechanism [18]. In contrast, subgroups of obesity, such as obesity without metabolic abnormalities, classified as ‘metabolically healthy obesity (MHO)’, have been proposed [17]. Predicting obesity-related risk is important because patients who require treatment can be identified.

Cluster analysis is a powerful tool for discovering subgroups of diseases and clarifying pathological conditions; therefore, it has recently been applied to various diseases, such as diabetes [19–22]. Regarding obesity, a cluster analysis using machine learning based on clinical variables revealed four stable metabolically distinct clusters [23]. Distinct subgroups were observed when sex-specific two-step cluster analysis was performed based on chronic medical conditions [24]. Some studies also performed subgroup analyses of metabolic health featuring obesity phenotypes. [21, 25, 26] However, most of these studies were based on cross-sectional analyses and biased conditions, and each cluster’s prognosis remains unclear, suggesting the necessity of studies which employ less-biased conditions to reveal the clinical heterogeneity of obesity and clarify the relationship between clinical characteristics and prognosis.

In the present study, we demonstrated new obesity subgroups based on unsupervised data-driven cluster analysis using a claims database, including health checkups and prognostic data.

## Methods

### Study populations

We used data from a claims database constructed by DeSC Healthcare, Inc., using standardised disease classifications and anonymous record linkage. This claims database includes monthly claims from all medical institutions and pharmacies, specific health checkups, and registry information in Japan submitted between April 2014 and February 2021, with approximately 3.44 million insured persons (approximately 2.74% of the Japanese population), comprised mainly of company employees and their family members. It has been reported that population structure of DeSC database is similar to that in Japan and DeSC database is suitable for the analysis of general Japanese population [27]. The DeSC database provides information on the beneficiaries, including encrypted personal identifiers, age, sex, International Classification of Diseases 10th revision (ICD-10) procedure and diagnostic codes, and the name, dose, and number of days for usage of the prescribed and/or dispensed drugs. All drugs were coded according to the therapeutic category based on the Kyoto Encyclopedia of Genes and Genomes (KEGG) drug and the Anatomical Therapeutic Chemical (ATC) classification of the European Pharmaceutical Market Research Association. An encrypted personal identifier was used to link the claims data from different hospitals, clinics, and pharmacies.

### Patients inclusion and characteristics analysis

We included patients with a BMI of ≥ 35 kg/m^2^ in the DeSC claims database. BMI, waist circumference, hemoglobin A1c (HbA1c), and estimated glomerular filtration rate (eGFR) data were collected during specific health checkups, which are obligatory annual health checkups conducted once yearly. Data on smoking history was also collected from health checkup records [28]. We used the validated technique as previously described [29] to conduct follow-up and longitudinal cohort surveys using administrative claims databases and specific health checkups to analyse such large and complex data.

### Prognosis analysis

Dialysis initiation was defined as the initial record of any dialysis-related code, and death was determined from the death flag in the DeSC database. The standardised mortality rate (SMR) was calculated using age- and sex-specific mortality rates obtained from ‘the 23rd Life Tables’ [30], which shows the results of the Population Census in 2020. SMR and 95% confidence interval were calculated as follows:

$$\text{SMR}=\frac{number\;of\;observed\;deaths}{number\;of\;expected\;deaths}$$

$$95\%\;\mathrm C\mathrm o\mathrm n\mathrm f\mathrm i\mathrm d\mathrm e\mathrm n\mathrm c\mathrm e\;\mathrm I\mathrm n\mathrm t\mathrm e\mathrm r\mathrm v\mathrm a\mathrm l=\text{SMR}\pm1.96\sqrt{\frac{SMR}{number\;of\;expected\;deaths}}$$

### Statistical analyses

Dimension reduction and cluster analysis were performed with the UMAP and the K-means algorithm. Briefly, the model variables were ICD-10, drug, and medical procedure codes. (Supplementary Fig. 1) The list of master codes for ICD-10, drug, and medical procedure are available upon the request, with permission by DeSC Healthcare, Inc. The presence or absence of each code is interpreted as a binary variable. For dimension reduction, uniform manifold approximation and projection (UMAP) was performed to reduce to two dimensions (n_components = 2) using the umap-learn library for Python (The python codes including parameters for UMAP are described in Supplementary data 1). The mathematical description of UMAP is available on the arxiv [31].

K-means clustering was performed with a k-value of 7 using the sklearn.cluster.KMeans from the scikit-learn library for Python. K-Means clustering is an unsupervised machine learning which categorizes the items into k groups of similarity [32]. The appropriate number of clusters was estimated from the elbow-plot (Supplementary Fig. 2), where the k-value around the “elbow” appeared in the plot is considered as optimal. In addition to the elbow plot, to validate the validity of the number of clusters, we conducted hierarchical clustering, showing that seven clusters were reproducible (Supplementary Data 2).

We performed a chi-square test in R version 4.1.1 to identify clinical features by comparing the distribution of each variable between the clusters. The chi-square test is a statistical method widely used to test a hypothesis about the distribution of a categorical variable frequently used in the previous studies [33, 34]. The formula for the chi-square test is as follows:

$${\upchi }^{2}=\sum \frac{{\left({O}_{i}- {E}_{i}\right)}^{2}}{{E}_{i}}$$

$$\chi^2=chi\;squared,\;O_i=observed\;value,\;E_i=expected\;value$$

The top 20 codes in each category were identified as cluster markers. Log-rank test was performed in IBM SPSS Statistics version 28.0.1 to analyse the renal/life prognosis. Log-rank test is a statistical method frequently used for survival analysis in the previous studies [35–37]. The log-rank statistics for a two-group case is as follows:

$$Log-rank\;statistics=\frac{\left(O_i-E_i\right)^2}{Var\left(O_i-E_i\right)}$$

$$Var\left({O}_{i}-{E}_{i}\right)= \frac{{n}_{1j}{n}_{2j}\left({m}_{1j}+{m}_{2j}\right)\left({n}_{1j}+{n}_{2j}-{m}_{1j}-{m}_{2j}\right)}{{\left({n}_{1j}+{n}_{2j}\right)}^{2}\left({n}_{1j}+{n}_{2j}-1\right)}$$

$$O_i=observed\;value,\;E_i=expected\;value$$

${m}_{ij}$= the numbers of subjects in the group (i) failing at that time.

${n}_{ij}$ = the numbers of subjects in the group (i) in the risk set at that time.

A *p*-value of less than 0.05 was considered significant in the prognosis analyses.

## Results

### Individuals with obesity were grouped into seven clusters

**Fig. 1:**
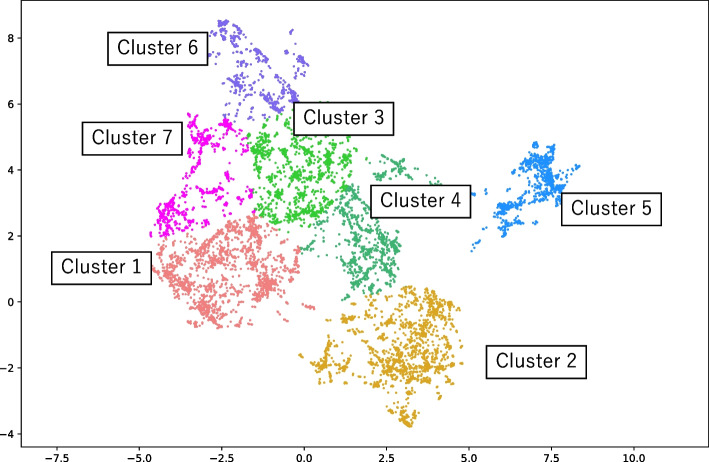
UMAP plots of all individuals with a BMI of ≥ 35 kg/m^2^

A total of 9,494 individuals with obesity (5,473 men and 4,021 women, mean age: 49.3 ± 13.6 years) were included in this study. The analyses by UMAP projection and k-means clustering revealed seven distinct clusters (Fig. 1), each with different clinical characteristics (Table 1). Furthermore, each cluster was characterised based on the ICD-10, drug, and medical procedure codes. (Supplementary Table 1–7). The names of each cluster shown in Table 1 are based on the clinical features indicated from the codes characteristically given to the clusters as listed in Supplementary Table 1–7. The results of the UMAP plot and the statistics of the codes given to the patients suggest that each cluster’s characteristics were unique. Cluster 1 (21.1%) was the most predominant and was characterised by hypertension, hyperlipidaemia, and diabetes. Cluster 2 (20.8%) was characterised by diabetes and retinopathy. Cluster 3 (16.0%) was characterised by allergic disease. Cluster 4 (11.9%) was characterised by ocular and allergic diseases. Cluster 5 (10.8%) was characterised by cardiovascular disease, diabetes, and surgery. Cluster 6 (10.2%) was characterised by the absence of medical follow-up. Cluster 7 (9.2%) was characterised by the presence of medical follow-ups but with rare ICD-10, drug, and medical procedure codes.

**Table 1:** Clinical characteristics of each cluster

Cluster	1	2	3	4	5	6	7
Characteristics	Hypertension/Dyslipidaemia/Diabetes	Diabetes/Retinopathy	Allergic disease	Eye disease/Allergic disease	Cardiovascular disease/Diabetes/Surgery	No medical follow up	Medical follow up
Patient number (%)	2000 (21.1)	1977 (20.8)	1518 (16.0)	1132 (11.9)	1029 (10.8)	968 (10.2)	870 (9.2)
Age (years) Mean (SD)	52.7 (12.9)	54.3 (12.9)	42.4 (11.6)	46.2 (12.2)	56.2 (12.9)	43.2 (12.2)	45.1 (12.8)
Male (%)	58.5	52.4	64.2	60.4	45.2	61.6	62.9
BMI (SD) (kg/m2)	37.2 (2.22)	37.1 (2.26)	37.0 (2.35)	36.9 (2.26)	37.1 (2.12)	37.1 (2.23)	36.9 (2.14)
Waist circumference (SD) (cm)	113.3 (8.58)	112.8 (9.53)	111.7 (8.98)	112.1 (8.91)	112.7 (8.73)	112.6 (8.10)	112.5 (7.99)
HbA1c (SD) (%)	6.26 (0.99)	6.57 (1.24)	5.90 (0.79)	5.95 (0.76)	6.19 (0.93)	5.87 (0.94)	5.93 (0.82)
eGFR (SD) (mL/min/1.73 m^2^)	73.0 (18.5)	74.0 (19.8)	81.9 (17.5)	78.0 (16.9)	72.4 (19.3)	81.5 (17.0)	81.0 (16.6)
Smoking (%)	24.7	19.6	28.4	25.9	21.5	29.8	26.2

Clusters 3, 6, and 7 were relatively young (42.4 ± 11.6, 43.2 ± 12.2, and 45.1 ± 12.8, respectively). Clusters 3, 4, 6, and 7 showed a male predominance (64.2, 60.4, 61.6, and 62.9%, respectively). Cluster 1 had the highest BMI (37.2 kg/m^2^), whereas Clusters 4 and 7 had the lowest (36.9). Cluster 1 showed the largest waist circumference (113.3 cm), whereas Cluster 3 showed the smallest (111.7 cm), followed by Cluster 4 (112.1 cm). Cluster 6 had the highest proportion of individuals with a smoking history (29.8%), followed by Cluster 3 (28.4%) and Cluster 4 (25.9%). Cluster 2 showed the highest HbA1c level (6.57%), whereas Cluster 6 showed the lowest (5.87%).

### Each cluster showed unique characteristics in the prescription

**Fig. 2:**
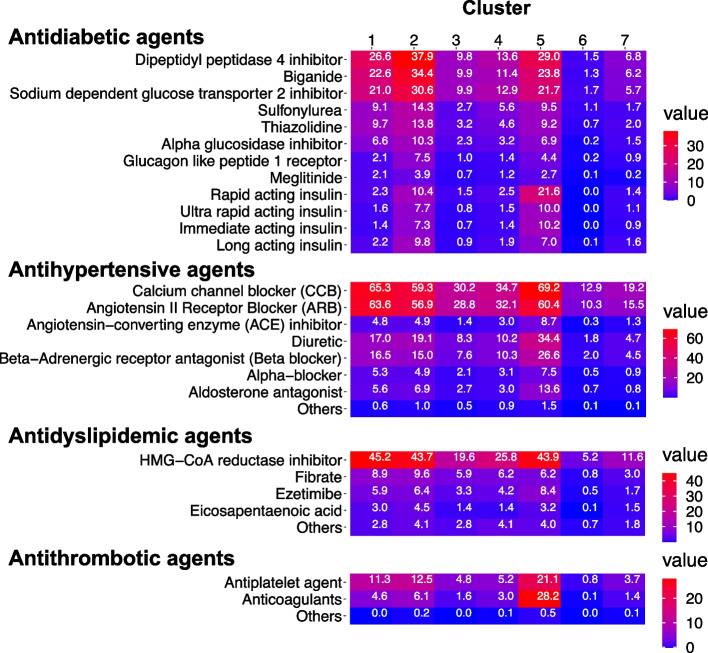
Heatmap depicting prescription for each subgroup. The proportion of individuals with medicine prescription records in each cluster is shown

We analysed the detailed prescriptions for each cluster (Fig. 2). The heatmap of each medication clearly showed that the individuals in Clusters 1, 2, and 5 were diabetic, hypertensive, and dyslipidaemic, respectively. Cluster 2 had the highest proportion of patients taking oral antidiabetic agents (52.5%), and Cluster 5 had the highest proportion of patients receiving insulin treatment (21.9%). Antihypertensive agents were prescribed for Clusters 1, 2, and 5. Anti-dyslipidaemic agents were prescribed for Clusters 1, 2, and 5. Antithrombotic agents were prescribed for Clusters 5, 2, and 1. Diuretics and beta-blockers were predominantly used in Cluster 5. These data indicate that Clusters 1, 2, and 5 were characterised as high-risk groups for cardiovascular diseases. Regarding surgery in Cluster 5, orthopaedic joint implants and spinal stenosis were predominantly described in the ICD-10 codes (Supplementary Table 5A). In contrast, antidiabetic and hypertensive agents were used less frequently in Clusters 3 and 4. Upper airway inflammation, allergic rhinitis, and asthma codes in ICD-10 were predominantly observed in Cluster 3 (Supplementary Table 9). Ophthalmic agents, such as ophthalmic cortisones, and ICD-10 codes similar to those of Cluster 3 were prescribed for Cluster 4.

### Each cluster exhibited different kidney prognosis and life expectancy

**Fig. 3:**
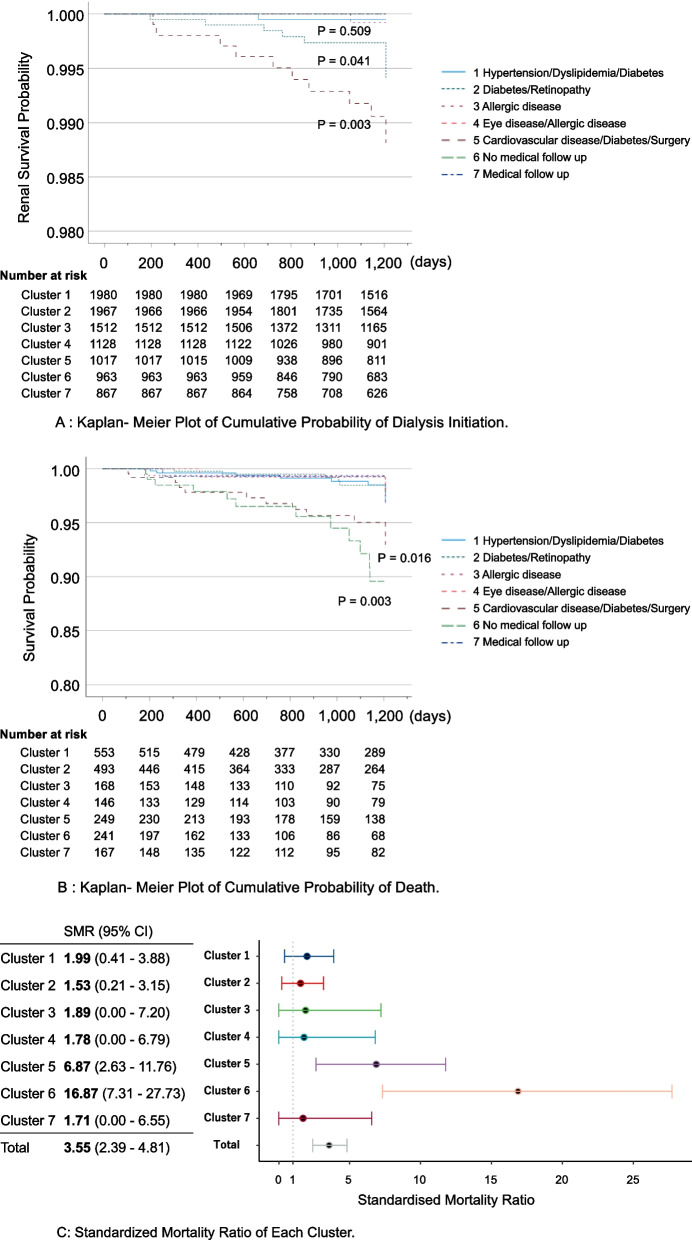
Kaplan–Meier Plots of the cumulative probability of dialysis initiation and death. Kaplan–Meier plots of the proportion of patients with outcome events after diagnosis of severe obesity (Body mass index ≥ 35 kg/m^2^) in each cluster are shown. The *p*-values were calculated in comparison with Cluster 7 using the log-rank test. **A** Kaplan–Meier plot of the cumulative probability of dialysis initiation. **B** Kaplan–Meier plot of the cumulative probability of death. The analysis includes only patients whose information about death is available. **C** Standardised mortality ratio for each cluster

Next, we analysed renal prognosis based on the initiation of dialysis for each cluster during the follow up period of 6 years after the diagnosis of obesity (Fig. 3A). Cluster 5 showed the highest risk for dialysis, followed by Cluster 2. The other clusters rarely showed a dialysis risk.

Furthermore, we analysed life expectancy (Fig. 3B). As expected, Cluster 5 showed a significantly higher mortality risk during the follow-up period of 6 years after the diagnosis of obesity. Notably, Cluster 6 had the highest mortality risk. Because the mean age at diagnosis differed among the clusters, we further analysed age- and sex-adjusted mortality (standardised mortality ratio). As shown in Fig. 3C, Cluster 6 showed the highest hazard ratio (HR) (HR: 16.87, 95% confidence interval [CI]: 7.31–27.73) compared with healthy controls, followed by Cluster 5 (HR: 6.87, 95% CI: 2.63–11.76) supporting the results in Fig. 3B. In contrast, Cluster 7 exhibited the lowest risk of complications and the best prognosis, which fits with the definition of MHO [17, 38]. The proportion was 9.2% in individuals with BMI of ≥ 35. We further analysed the proportions of these clusters in different BMIs using this claimed database by the same method. Interestingly, the proportion of the cluster characterized by the presence of medical follow-ups but with rare ICD-10, drug, and medical procedure codes in the individuals with BMIs of 25–30 (N = 10,574, seven clusters) and 30–35 (N = 45,932, seven clusters) were 17.5% and 11.0%, respectively (Supplementary Fig. 3).

### Healthcare costs of each cluster

Cluster 5 showed the highest expenditure, whereas Cluster 6 showed the lowest throughout the 1,800 days after diagnosis. (Supplementary Fig. 4).

## Discussion

In the present study, we identified seven clusters of obese individuals with distinct clinical characteristics and prognoses. Patients were grouped into seven clusters characterised by complications, such as diabetes, hypertension, dyslipidaemia, and allergic, respiratory, ocular, and cardiovascular diseases. In addition, renal survival and life prognosis differed among the clusters, suggesting that appropriate medical care/management greatly varies depending on obese patients’ clinical conditions. Cluster 5 and 6 showed significantly higher mortality rates compared with that of general population. Notably, Cluster 6, with a rare history of medical checkups/treatment, showed the poorest prognosis.

The distance between dots in UMAP plots reflected similarities in each cluster (Fig. 1). Specifically, Cluster 1, 2 and 5 exhibited the similar profiles regarding the prescription of antidiabetic agents, antihypertensive agents, and antidyslipidemic agents (Fig. 2). On the other hand, the surgery-related codes were predominantly given to Cluster 5 (Supplementary Table 5A—5C), which also uniquely showed its high prevalence of cardiovascular disease. Cluster 3 and 4 had common clinical similarities regarding the allergic diseases, BMI, waist circumference, and the high proportion of smokers. On the other hand, the dissimilarity lies in the proportions of individuals who underwent ophthalmic procedures. Cluster 6 showed its unique characteristics of a rare history of medical checkups/treatment (Supplementary Table 6A—6C). Also, Cluster 7 exhibited the lowest prevalence of complications, which is quite different to other clusters (Supplementary Table 7A—7C).

Cluster analyses of populations with obesity have been performed in several studies. However, most studies were conducted on a relatively small number of patients or based on data from health and nutrition surveys, which may include various biases. The selection of factors for analysis can be influenced by the knowledge, thoughts, and belief of the researchers who design and conduct the analyses, leading to biased classification. In the present cross-sectional study, we used a claims database consisting of comprehensive medical information; therefore, there was minimum selection bias regarding the dataset’s characteristics. This study aimed to clarify the clinical heterogeneity of the obese population-based on an unbiased classification by study design which considers unknown factors regarding the clinical characteristics of individuals with obesity. In addition, we used an integrated claims database that included health checkup data and information about death to enable us to replicate clinical studies by the modern cluster classification and analyse the associations of the clinical characteristics with prognosis in obese individuals, indicating that we demonstrated the essential subgroups using different approaches. Furthermore, our study showed a result of a comprehensive analysis while these different conditions were generally discussed separately in previous reports. Also, we showed the possible relationships between the clinical characteristics and prognosis in obese population, which has not been addressed in the previous studies [23–26].

There were three clusters with a high proportion of patients with diabetes and increased HbA1c levels. The subgroups of obesity characterized by a high risk of diabetes were also indicated in the previous study [23]. Clusters 1 and 2 showed similar characteristics in terms of prescription, with a high proportion of patients taking oral antidiabetic, antihypertensive, and antidyslipidaemic agents. Cluster 1 exhibited a considerably high prevalence of hypertension (Supplementary Table 1A—1C), while Cluster 2 showed uniquely high proportion of the individuals who underwent ophthalmic and/or antidiabetic treatments (Supplementary Table 2A—2C).

In addition, Cluster 2 had a relatively higher proportion of patients with retinopathy, suggesting the presence of progressive microangiopathy (Supplementary Table 8) with higher HbA1c levels. These data explain the renal prognosis deteriorated in Cluster 2 compared with Cluster 1. Cluster 1 showed the largest waist circumference, followed by Clusters 2 and 5, suggesting that increased visceral fat, as a metabolic syndrome, may be associated with the condition of increased diabetes, hypertension, and dyslipidaemia in Cluster 1. This finding is in accordance with the previous study which found the subgroup of obesity characterised by high BMI, waist-hip ratio, and dyslipidemia [25].

In contrast, Cluster 5 had a high proportion of patients receiving insulin treatment, suggesting the presence of severe diabetes with long disease duration. The most frequent use of antihypertensive, antidyslipidaemic, and antithrombotic agents was observed in Cluster 5, suggesting the presence of progressive macroangiopathy. Furthermore, renal prognosis and mortality were poor in Cluster 5, suggesting that more aggressive interventions, including bariatric surgery, may be necessary. Interestingly, this cluster had a considerably high proportion of patients who underwent orthopaedic surgeries, such as joint replacement, suggesting an association between arthropathy and macroangiopathy. In the surgery cluster, orthopedic joint implants and spinal stenosis were predominant, suggesting the orthopedic deterioration due to overweight. Although precise relationship is unclarified, these results indicate the possibility that physical issues such as related-sarcopenia caused by reduced physical activity worsened their prognosis. Differences in the clinical characteristics of microangiopathy and macroangiopathy among these clusters suggest different underlying pathological conditions. Sarcopenic obesity has been reported to be associated with increased risks of metabolic diseases [39–41] and cardiovascular diseases [42, 43].

Clusters 3 and 4 had a high proportion of patients with allergic diseases, including asthma, upper airway inflammation, and allergic rhinitis (Supplementary Table 3A—4C). Obesity is well known as a major risk factor for asthma including allergic diseases [44–47]. In addition, Clusters 3 and 4 had a higher proportion of smokers, which may be associated with these conditions. Interestingly, Clusters 3 and 4 showed relatively smaller waist circumferences than the other clusters, suggesting subcutaneous fat-dominant-type obesity. Subcutaneous obesity has been recently reported to be associated with the increased risk of asthma in patients with obesity [48, 49]. Different adipokine and chemokine profiles in patients with subcutaneous obesity from visceral obesity may reflect the risk of allergic diseases and asthma. Notably, diabetes, hypertension, and dyslipidaemia were relatively rare in Clusters 3 and 4. Cluster 4 had a higher proportion of individuals diagnosed with ocular diseases, such as astigmatism and conjunctivitis (Supplementary Table 9), and those who underwent ophthalmic procedures than Cluster 3; however, these clusters show a similar pathological background. These results indicate that prohibition of smoking may be especially important in these clusters regarding their allergies. The complications such as allergic disease (Cluster 3 and 4) and cardiovascular diseases (Cluster 5) were almost exclusive, suggesting a presence of different pathological conditions. These findings regarding Cluster 3, 4, and 5 have not been reported in the previous studies based on cluster analysis of obesity, which mainly focused on the biomedical markers and widely-known metabolic comorbidities of obesity [23–26].

Cluster 7 exhibited the lowest risk of complications and the best prognosis, which fit the definition of MHO regarding their clinical condition and its favorable prognosis [17, 38]. The MHO subgroup found in this study is in accordance with the literature regarding the subtypes of obesity [23]. Notably, individuals in Cluster 7 visited hospitals and underwent medical examinations and treatments, if necessary. The present study clearly demonstrated the existence of healthy individuals with obesity with a prevalence of 9.2% under population-based conditions. The proportions of the clusters considered as MHO in the population with BMIs of 25–30 and 30–35 were 17.5% and 11.0%, respectively, indicating the association between a higher BMI and a decrease in the proportion of MHO. In contrast, Cluster 6, which had patients with almost no history of medical checkups/treatment, demonstrated the highest mortality rate among the clusters, suggesting the possible relationship between medical examinations and prognosis as an issue for future research. The presence of a subgroup which had a rare history of medical interventions have not been revealed in the previous studies since comprehensive claims data has not been used for cluster analysis. Although it is speculated that the increased mortality is associated with obesity in this cluster, given that the cause of death cannot be identified from the DeSC database, the relationship between the poor compliance and the risk of death remains unclear. The cases of death may include not only the results of obesity complications but also the events out of medical management such as accidents and suicides. Further studies are necessary to clarify the relationship between medical compliance and increased mortality; however, these data suggest the potential for reducing the mortality rate through the promotion of medical check-ups and appropriate interventions.

Identifying diagnostic markers for poor prognosis subgroups enables appropriate patients for aggressive medical interventions. Metabolic surgery has not been widespread in Japan, and the population that could benefit from this surgery remains under debate. This study suggests that the need for metabolic surgery in obese populations could vary depending on individuals’ clinical conditions. Intensive intervention would be beneficial for individuals in the subgroups with poor prognosis rather than MHO population, suggesting the possibility of more efficient allocation of medical resources by selecting appropriate targets for medical intervention. In particular, the economic benefits of metabolic surgery can be expected in Cluster 5, which was characterized with a notably high risk of orthopaedic issues. In contrast, individuals in Cluster 7 (individuals with MHO) would gain little benefit from metabolic surgery due to their favorable prognosis without intensive treatments.

This study had several limitations. First, we demonstrated clear clinical heterogeneity and prognosis in Japanese individuals with obesity; however, racial differences may exist. Therefore, similar analyses in different races are necessary. Second, we used claimed data in which the individuals conducted health checkups, suggesting a presence of selection bias. Also, the patients not registered on the claims database are not included in this study and prescription adherence and the possibility of misdiagnoses cannot be assessed from the data. Third, the results from this study do not represent causal evidence. Therefore, the mechanism that underlies the association between poor medical compliance and prognosis remains unclear and should be addressed in the future studies. Since we have not conducted intensive medical intervention to the targeted subgroups, a prospective study should be performed in order to validate the effectiveness of intensive medical intervention. Regarding the constrains for the validity, there is uncertainty regarding the effect on clustering due to the selection of two dimensions for the reduced dimension space (n_components = 2) in terms of visualization. Due to the nature of dimension reduction, the result of visualization could be different depending on the parameters for the analysis. Also, the optimal/appropriate number of the clusters are subject to the methodology of dimension reduction and clustering. Therefore, further studies using other cohorts will be required to ensure the validity.

Additionally, we cannot rule out the possibility of over-fitting in this model since we compared the observed clusters using the same training data. Further investigation with another cohort is necessary to ensure the reliability of the patterns including the representative clinical characteristics of each cluster observed in this study. Nevertheless, this new subgroup of individuals with obesity gives an important clue to optimize the appropriate interventions. This study provides novel evidence of clinical heterogeneity using less biased information, and offers new insights regarding the patient selection for medical checkups and intensive treatments.

## Conclusions

In conclusion, we identified seven subgroups of individuals with obesity using population-based data-driven cluster analysis. We clearly demonstrated important target subgroups for intervention as well as a metabolically healthy obesity group.

### Supplementary Information

**Additional file 1: Supplementary Table 1A.** ICD-10 codes for Cluster 1. **Supplementary Table 1B.** Drug codes for Cluster 1. **Supplementary Table 1C.** Medical procedure codes for Cluster 1. **Supplementary Table 2A.** ICD-10 codes for Cluster 2. **Supplementary Table 2B.** Drug codes for Cluster 2. **Supplementary Table 2C.** Medical procedure codes for Cluster 2. **Supplementary Table 3A.** ICD-10 codes for Cluster 3. **Supplementary Table 3B.** Drug codes for Cluster 3. **Supplementary Table 3C.** Medical procedure codes for Cluster 3. **Supplementary Table 4A.** ICD-10 codes for Cluster 4. **Supplementary Table 4B.** Drug codes for Cluster 4. **Supplementary Table 4C.** Medical procedure codes for Cluster 4. **Supplementary Table 5A.** ICD-10 codes for Cluster 5. **Supplementary Table 5B.** Drug codes for Cluster 5. **Supplementary Table 5C.** Medical procedure codes for Cluster 5. **Supplementary Table 6A.** ICD-10 codes for Cluster 6. **Supplementary Table 6B.** Drug codes for Cluster 6. **Supplementary Table 6C.** Medical procedure codes for Cluster 6. **Supplementary Table 7A.** ICD-10 codes for Cluster 7. **Supplementary Table 7B.** Drug codes for Cluster 7. **Supplementary Table 7C.** Medical procedure codes for Cluster 7. **Supplementary Table 8.** ICD-10, drug, and medical procedure codes indicating retinopathy in Cluster 2. **Supplementary Table 9A.** ICD-10 codes indicating ocular diseases in Clusters 3 and 4. **Supplementary Table 9B.** Drug codes indicating ocular diseases in Clusters 3 and 4. **Supplementary Table 9C.** Medical procedure codes indicating ocular diseases in Clusters 3 and 4. **Supplementary Fig. 1.** Flowchart of the study. **Supplementary Fig. 2.** Elbow-Plot for K-means clustering **Supplementary Fig. 3.** Association between BMI and the proportion of the individuals with MHO. **Supplementary Fig. 4.** Health care fee of each cluster. **Supplementary Fig. 5.** UMAP plots of all individuals with BMI 30-35. **Supplementary Data 1.** Python Code for UMAP Reduction and K-means Clustering. **Supplementary Data 2.** Hierarchical Clustering.

## Data Availability

The list of master codes for ICD-10, drug, and medical procedure are available upon the request to saki.t1017@gmail.com, with permission by DeSC Healthcare, Inc. Group-level data are available in DeSC database.
