# Supplementary material for: Novel subgroups of obesity and their association with outcomes: a data-driven cluster analysis
Source: BMC Public Health. 2024 Jan 9;24:124. doi: 10.1186/s12889-024-17648-1 (PMC10775568; doi:10.1186/s12889-024-17648-1)

Supplementary Table 1A. ICD-10 codes for Cluster 1.

class	ident_pct	others_pct	Description
ICD10_H522	16.65	47.26447825	Astigmatism
**ICD10_I10**	**83.2**	**54.00320256**	**Essential (primary) hypertension**
ICD10_J060	15.9	29.42353883	Acute laryngopharyngitis
ICD10_J019	6.05	16.37309848	Acute sinusitis, unspecified
**ICD10_E785**	**57.05**	**42.35388311**	**Hyperlipidaemia, unspecified**
ICD10_H524	2.3	9.32746197	Presbyopia
ICD10_E143	3.25	10.64851882	Unspecified diabetes mellitus with ophthalmic complications
ICD10_H109	7.45	16.3997865	Conjunctivitis, unspecified
ICD10_H041	4.95	12.74352816	Other disorders of lacrimal gland
**ICD10_E14**	**48.05**	**36.01547905**	**Unspecified diabetes mellitus**
ICD10_E790	31.75	21.45716573	Hyperuricaemia without signs of inflammatory arthritis and tophaceous disease
ICD10_J209	40.5	52.54870563	Acute bronchitis, unspecified
ICD10_J101	10.25	19.25540432	Influenza with other respiratory manifestations, other influenza virus identified
ICD10_J304	40.25	52.10835335	Allergic rhinitis, unspecified
**ICD10_E780**	**33.6**	**23.27195089**	**Pure hypercholesterolaemia**
ICD10_H269	4.2	11.23565519	Cataract, unspecified
ICD10_J039	9.65	18.2812917	Acute tonsillitis, unspecified
ICD10_H101	15.1	24.67307179	Acute atopic conjunctivitis
ICD10_J069	30.95	42.08700294	Acute upper respiratory infection, unspecified
ICD10_J029	29.2	40.01868161	Acute pharyngitis, unspecified

ident_pct: proportion of individuals having a record of the code in the selected cluster.
others_pct: proportion of individuals having a record of the code in all other clusters.

Table is sorted in ascending order by the P-value (P-value < 0.05).
Rows in bold type indicate clinical characteristics of that cluster.

Supplementary Table 1B. Drug codes for Cluster 1.

class	ident_pct	others_pct	Description
**Drug_2149**	**67.5**	**40.40565786**	**Antihypertensives, Others**
Drug_1315	14.6	36.42914331	Ophthalmic cortisones
Drug_1319	28.25	50.65385642	Ophthalmic agents, Others
**Drug_2189**	**48.25**	**29.8372031**	**Hyperlipidemia agents, Others**
Drug_1119	1.05	12.91700027	General anesthetics, Others
Drug_2171	53.5	34.85455031	Coronary dilators
Drug_3327	24.2	42.83426741	Antiplasmins
Drug_1141	47.85	65.33226581	Anilines
Drug_1229	1.5	11.4091273	Skeletal muscle relaxants, Others
Drug_6132	53.4	69.46890846	Cephem antibioitics
Drug_3319	23.55	39.87189752	Blood substitutes, Others
Drug_3334	3.05	13.89111289	Heparins
Drug_3311	33.5	50.26688017	Physiological salines
Drug_7131	5.85	17.06698692	Purified water
Drug_2451	4.7	15.05204163	Epinephrines
Drug_2221	1.5	9.727782226	Ephedrines and Ephedra herbs
Drug_2399	32.35	47.26447825	Digestive organ agents, Miscellaneous
Drug_2233	41.6	56.28502802	Cysteines
Drug_6250	20.2	33.7469976	Antivirals
Drug_3929	4.65	13.66426475	Antidotes, Others

ident_pct: proportion of individuals having a record of the code in the selected cluster.
others_pct: proportion of individuals having a record of the code in all other clusters.

Table is sorted in ascending order by the P-value (P-value < 0.05).
Rows in bold type indicate clinical characteristics of that cluster.

Supplementary Table 1C. Medical procedure codes for Cluster 1.

class	ident_pct	others_pct	Description
Proc_D263	15.05	47.98505471	Corrective vision test
Proc_D261	11.4	43.63490793	Refraction test
Proc_D264	16.2	49.11929544	Precision tonometry
Proc_D255	17.05	48.03843074	Precision fundus examination (unilateral)
Proc_D265	7.75	36.18895116	Precision fundus examination (unilateral)
Proc_D273	18.7	46.47718175	Slit lamp microscopy (anterior segment)
Proc_B0	84	57.52602082	Treatment for specific diseases
Proc_D7	98.75	79.02321857	Blood chemistry tests
Proc_D5	97.35	78.91646651	Blood morphology/function test
Proc_D400	98.6	81.38510809	Blood sampling (per day)
Proc_D256	8.75	28.50280224	Fundus camera photography
Proc_D12	47.65	69.20202829	Infectious disease immunological test
Proc_D257	7.45	25.90072058	Slit-lamp microscopy (anterior and posterior segments)
Proc_D419	26.7	45.58313317	Sample collection
Proc_L8	0.65	11.68935148	Closed circulation general anesthesia with mask or endotracheal intubation
Proc_D26	99.95	89.76514545	Specimen test judgment
Proc_B8	6.05	19.88257272	Drug management instruction fee
Proc_A234	9.45	24.53963171	Additional medical safety measures (first day of hospitalization)
Proc_J97	10.95	26.38110488	Nasal Medical procedure
Proc_D11	4.75	17.69415532	Immunohematological test

ident_pct: proportion of individuals having a record of the code in the selected cluster.
others_pct: proportion of individuals having a record of the code in all other clusters.

Table is sorted in ascending order by the P-value (P-value < 0.05).
Rows in bold type indicate clinical characteristics of that cluster.

Supplementary Table 2A. ICD-10 codes for Cluster 2.

class	ident_pct	others_pct	Description
**ICD10_H522**	**90.38947901**	**27.77703871**	**Astigmatism**
**ICD10_E143**	**29.69145169**	**3.671677531**	**Unspecified diabetes mellitus with ophthalmic complications**
**ICD10_H269**	**27.46585736**	**5.095117733**	**Cataract, unspecified**
**ICD10_Z961**	**16.94486596**	**2.367965944**	**Presence of intraocular lens**
**ICD10_H524**	**19.27162367**	**4.842357323**	**Presbyopia**
**ICD10_H409**	**13.8593829**	**2.740454969**	**Glaucoma, unspecified**
**ICD10_H041**	**23.36874052**	**7.87548224**	**Other disorders of lacrimal gland**
**ICD10_E14**	**57.4102175**	**33.59052814**	**Unspecified diabetes mellitus**
**ICD10_H400**	**14.46636318**	**3.312491686**	**Glaucoma suspect**
**ICD10_E11**	**40.16186141**	**19.72861514**	**Non-insulin-dependent diabetes mellitus**
**ICD10_H353**	**10.11633789**	**1.928961022**	**Degeneration of macula and posterior pole**
ICD10_H109	26.3024785	11.41412798	Conjunctivitis, unspecified
**ICD10_I10**	**75.92311583**	**56.00638553**	**Essential (primary) hypertension**
**ICD10_H350**	**7.587253414**	**1.23719569**	**Background retinopathy and retinal vascular changes**
**ICD10_E142**	**11.38088012**	**3.059731276**	**Unspecified diabetes mellitus with renal complications**
**ICD10_E785**	**59.53464846**	**41.74537715**	**Hyperlipidaemia, unspecified**
**ICD10_H259**	**7.738998483**	**1.689503791**	**Senile cataract, unspecified**
ICD10_H168	10.41982802	3.086337635	Other keratitis
**ICD10_H438**	**8.447142135**	**2.128508714**	**Other disorders of vitreous body**
ICD10_H531	11.83611533	4.097379274	Subjective visual disturbances

ident_pct: proportion of individuals having a record of the code in the selected cluster.
others_pct: proportion of individuals having a record of the code in all other clusters.

Table is sorted in ascending order by the P-value (P-value < 0.05).
Rows in bold type indicate clinical characteristics of that cluster.

Supplementary Table 2B. Drug codes for Cluster 2.

class	ident_pct	others_pct	Description
**Drug_1319**	**79.76732423**	**37.03605162**	**Ophthalmic agents, Others**
**Drug_3969**	**49.16540212**	**22.03006519**	**Antidiabetic agents, Others**
**Drug_3962**	**32.67577137**	**12.85087136**	**Biguanides**
**Drug_1311**	**7.132018209**	**0.798190768**	**Mydriatic agents**
Drug_1313	8.447142135	1.556471997	Ophthalmic local anesthetics
**Drug_2149**	**60.64744562**	**42.2908075**	**Antihypertensives, Others**
Drug_7290	29.64087001	15.85738992	Intracorporeal diagnostic agents, Miscellaneous
Drug_1315	44.61305008	28.46880404	Ophthalmic cortisones
**Drug_2189**	**46.68689934**	**30.30464281**	**Hyperlipidemia agents, Others**
Drug_3961	14.26403642	5.42769722	Sulfonylureas
Drug_2492	12.99949418	4.908873221	Pancreatic hormones
**Drug_2171**	**48.96307537**	**36.10482905**	**Coronary dilators**
Drug_2499	8.143651998	3.019821737	Hormones, Miscellaneous
Drug_2221	3.237228123	9.245709725	Ephedrines and Ephedra herbs
Drug_1119	5.260495701	11.77331382	General anesthetics, Others
Drug_1229	4.45118867	10.60263403	Skeletal muscle relaxants, Others
Drug_3999	24.63328275	16.56245843	Other agents affecting metabolism, Miscellaneous
Drug_2491	4.501770359	1.4899561	Circulatory hormones
Drug_2134	1.972685888	0.372489025	Carbonic anhydrase inhibitors
Drug_1214	58.16894284	49.06212585	Xylidines

ident_pct: proportion of individuals having a record of the code in the selected cluster.
others_pct: proportion of individuals having a record of the code in all other clusters.

Table is sorted in ascending order by the P-value (P-value < 0.05).
Rows in bold type indicate clinical characteristics of that cluster.

Supplementary Table 2C. Medical procedure codes for Cluster 2.

class	ident_pct	others_pct	Description
Proc_D255	97.6226606	26.75269389	Precision fundus examination (unilateral)
Proc_D256	65.85735964	13.42290808	Fundus camera photography
Proc_D257	61.86140617	11.53385659	Slit-lamp microscopy (anterior and posterior segments)
Proc_D261	90.23773394	22.80164959	Refraction test
Proc_D263	98.53313101	25.92789677	Corrective vision test
Proc_D264	98.98836621	27.24491153	Precision tonometry
Proc_D265	78.40161861	17.52028735	Precision fundus examination (unilateral)
Proc_D273	82.04350025	29.73260609	Slit lamp microscopy (anterior segment)
Proc_D260	23.21699545	4.962085939	Quantitative visual field test (unilateral)
Proc_D279	12.54425898	1.583078356	Corneal endothelial cell microscopy
Proc_D262	20.33383915	4.908873221	Adjustment test
**Proc_K282**	**9.863429439**	**0.944525742**	**Cataract surgery**
Proc_D1	32.0687911	13.11693495	Urinary special substance qualitative and quantitative test
Proc_D269	7.738998483	0.74497805	Protrusion measurement
**Proc_L6**	**6.423874557**	**0.585339896**	**Retrobulbar anesthesia and Conduction anesthesia of the face and head and neck**
**Proc_C101**	**12.64542236**	**3.365704403**	**Guidance for self-injection at home**
**Proc_C150**	**9.205867476**	**2.101902355**	**Blood glucose self-monitoring device**
Proc_D8	60.19221042	42.33071704	Endocrinological examination
Proc_D7	93.42438037	80.48423573	Blood chemistry tests
Proc_D400	93.93019727	82.66595716	Blood sampling (per day)

ident_pct: proportion of individuals having a record of the code in the selected cluster.
others_pct: proportion of individuals having a record of the code in all other clusters.

Table is sorted in ascending order by the P-value (P-value < 0.05).
Rows in bold type indicate clinical characteristics of that cluster.

Supplementary Table 3A. ICD-10 codes for Cluster 3.

class	ident_pct	others_pct	Description
**ICD10_J209**	**75.82345191**	**45.09779338**	**Acute bronchitis, unspecified**
**ICD10_J304**	**73.1884058**	**45.12286861**	**Allergic rhinitis, unspecified**
**ICD10_J029**	**59.15678524**	**33.66349047**	**Acute pharyngitis, unspecified**
**ICD10_J069**	**61.06719368**	**35.68204614**	**Acute upper respiratory infection, unspecified**
ICD10_H522	19.6969697	44.83450351	Astigmatism
**ICD10_J101**	**33.53096179**	**14.28034102**	**Influenza with other respiratory manifestations, other influenza virus identified**
ICD10_J060	43.47826087	23.35757272	Acute laryngopharyngitis
ICD10_I10	42.49011858	63.51554664	Essential (primary) hypertension
**ICD10_J019**	**26.15283267**	**11.92326981**	**Acute sinusitis, unspecified**
ICD10_A099	42.55599473	24.88716148	Gastroenteritis and colitis of unspecified origin
ICD10_J459	44.1370224	27.2442327	Asthma, unspecified
ICD10_J00	31.88405797	17.72818455	Acute nasopharyngitis [common cold]
ICD10_J039	27.20685112	14.41825476	Acute tonsillitis, unspecified
ICD10_E14	24.57180501	41.2111334	Unspecified diabetes mellitus
ICD10_K291	27.60210804	15.1328987	Other acute gastritis
ICD10_H269	1.646903821	11.29638917	Cataract, unspecified
ICD10_E143	1.317523057	10.56920762	Unspecified diabetes mellitus with ophthalmic complications
ICD10_J111	18.37944664	8.864092277	Influenza with other respiratory manifestations, virus not identified
ICD10_K053	66.60079051	52.89618857	Chronic periodontitis
ICD10_Z961	0.592885375	6.318956871	Presence of intraocular lens

ident_pct: proportion of individuals having a record of the code in the selected cluster.
others_pct: proportion of individuals having a record of the code in all other clusters.

Table is sorted in ascending order by the P-value (P-value < 0.05).
Rows in bold type indicate clinical characteristics of that cluster.

Supplementary Table 3B. Drug codes for Cluster 3.

class	ident_pct	others_pct	Description
**Drug_2233**	**78.98550725**	**48.28234704**	**Cysteines**
**Drug_3327**	**63.89986825**	**34.15245737**	**Antiplasmins**
**Drug_1141**	**86.16600791**	**56.98345035**	**Anilines**
**Drug_6149**	**67.25955204**	**38.92928786**	**Acting mainly on gram-positive bacteria and mycoplasma, Others**
**Drug_6250**	**52.76679842**	**26.73019057**	**Antivirals**
**Drug_4490**	**78.52437418**	**52.10631896**	**Allergic agents, Miscellaneous**
**Drug_2229**	**59.68379447**	**33.85155466**	**Antitussives, Others**
Drug_2249	37.41765481	18.39267803	Antitussives and expectorants, Others
Drug_2316	56.19235837	33.7888666	Probiotics
Drug_6241	63.83399209	40.83500502	Pyridonecarboxylic acids
Drug_2223	46.31093544	25.67703109	Dextromethorphans
Drug_2239	41.43610013	23.08174524	Expectorants, Others
Drug_2260	41.43610013	23.08174524	Gargles
Drug_1180	48.94598155	29.62637914	Common cold drugs
Drug_2399	60.93544137	40.9227683	Digestive organ agents, Miscellaneous
Drug_1149	92.95125165	77.68304915	Antipyretics and analgesics, anti-inflammatory agents, Others
Drug_2259	36.62714097	20.78736209	Bronchodilators, Others
Drug_2329	85.90250329	69.22016048	Peptic ulcer agents, Others
Drug_2149	30.69828722	49.04714142	Antihypertensives, Others
Drug_3969	14.22924901	30.24072217	Antidiabetic agents, Others

ident_pct: proportion of individuals having a record of the code in the selected cluster.
others_pct: proportion of individuals having a record of the code in all other clusters.

Table is sorted in ascending order by the P-value (P-value < 0.05).
Rows in bold type indicate clinical characteristics of that cluster.

Supplementary Table 3C. Medical procedure codes for Cluster 3.

class	ident_pct	others_pct	Description
Proc_D419	69.89459816	36.22116349	Sample collection
Proc_D263	14.82213439	46.03811434	Corrective vision test
Proc_D255	16.46903821	46.27632899	Precision fundus examination (unilateral)
Proc_D264	17.12779974	46.95336008	Precision tonometry
Proc_D261	12.77997365	41.42427282	Refraction test
Proc_D12	88.07641634	60.20561685	Infectious disease immunological test
Proc_D265	8.893280632	34.25275827	Precision fundus examination (unilateral)
Proc_D257	4.808959157	25.2883651	Slit-lamp microscopy (anterior and posterior segments)
Proc_D256	6.916996047	27.65797392	Fundus camera photography
**Proc_J114**	**37.22002635**	**18.4553661**	**Nebulizer**
Proc_D273	23.91304348	43.80641926	Slit lamp microscopy (anterior segment)
**Proc_J97**	**37.22002635**	**20.44884654**	**Nasal Medical procedure**
**Proc_J99**	**21.47562582**	**10.09277834**	**Indirect laryngoscopic laryngeal procedure**
Proc_D26	99.53886693	90.45887663	Specimen test judgment
Proc_D208	32.21343874	48.67101304	Electrocardiogram
Proc_F100	79.0513834	64.36810431	Prescription fee
Proc_J98	14.75625823	6.67001003	Oral and pharyngeal procedures
Proc_L8	2.108036891	10.7447342	Closed circulation general anesthesia with mask or endotracheal intubation
Proc_B8	8.036890646	18.66850552	Drug management instruction fee
Proc_F400	98.94598155	91.81293882	Prescription recipe fee

ident_pct: proportion of individuals having a record of the code in the selected cluster.
others_pct: proportion of individuals having a record of the code in all other clusters.

Table is sorted in ascending order by the P-value (P-value < 0.05).
Rows in bold type indicate clinical characteristics of that cluster.

Supplementary Table 4A. ICD-10 codes for Cluster 4.

class	ident_pct	others_pct	Description
**ICD10_J019**	**32.33215548**	**11.74360201**	**Acute sinusitis, unspecified**
**ICD10_H522**	**65.45936396**	**37.47907199**	**Astigmatism**
**ICD10_J060**	**48.67491166**	**23.58287491**	**Acute laryngopharyngitis**
**ICD10_J304**	**73.32155477**	**46.40038268**	**Allergic rhinitis, unspecified**
**ICD10_H101**	**40.28268551**	**20.27027027**	**Acute atopic conjunctivitis**
ICD10_H109	28.44522968	12.62855776	Conjunctivitis, unspecified
**ICD10_J209**	**69.16961131**	**47.41688591**	**Acute bronchitis, unspecified**
**ICD10_J039**	**30.4770318**	**14.56589333**	**Acute tonsillitis, unspecified**
**ICD10_J029**	**55.74204947**	**35.3025592**	**Acute pharyngitis, unspecified**
ICD10_J329	26.32508834	12.04257355	Chronic sinusitis, unspecified
**ICD10_J069**	**56.44876325**	**37.47907199**	**Acute upper respiratory infection, unspecified**
**ICD10_J040**	**11.48409894**	**3.719205932**	**Acute laryngitis**
**ICD10_J101**	**28.97526502**	**15.7856972**	**Influenza with other respiratory manifestations, other influenza virus identified**
**ICD10_J459**	**43.90459364**	**28.05548912**	**Asthma, unspecified**
ICD10_H609	12.63250883	5.094475006	Otitis externa, unspecified
ICD10_A099	40.01766784	26.04640038	Gastroenteritis and colitis of unspecified origin
ICD10_K053	68.46289753	53.27672806	Chronic periodontitis
ICD10_L309	49.46996466	34.84812246	Dermatitis, unspecified
ICD10_J40	25.795053	15.11600096	Bronchitis, not specified as acute or chronic
ICD10_J00	30.12367491	18.61994738	Acute nasopharyngitis [common cold]

ident_pct: proportion of individuals having a record of the code in the selected cluster.
others_pct: proportion of individuals having a record of the code in all other clusters.

Table is sorted in ascending order by the P-value (P-value < 0.05).
Rows in bold type indicate clinical characteristics of that cluster.

Supplementary Table 4B. Drug codes for Cluster 4.

class	ident_pct	others_pct	Description
**Drug_1315**	**62.80918728**	**27.63692896**	**Ophthalmic cortisones**
Drug_1329	43.19787986	18.2731404	Otic and nasal agents, Others
**Drug_1319**	**71.81978799**	**42.43004066**	**Ophthalmic agents, Others**
**Drug_1325**	**28.09187279**	**10.52379813**	**Otolaryngologic antibiotics**
**Drug_6149**	**65.45936396**	**40.48074623**	**Acting mainly on gram-positive bacteria and mycoplasma, Others**
Drug_3327	59.89399293	36.06792633	Antiplasmins
**Drug_4490**	**77.38515901**	**53.4800287**	**Allergic agents, Miscellaneous**
Drug_7131	29.50530035	12.70031093	Purified water
Drug_6250	49.29328622	28.4022961	Antivirals
Drug_2233	73.0565371	50.50227218	Cysteines
Drug_6241	62.63250883	42.05931595	Pyridonecarboxylic acids
Drug_2223	45.49469965	26.74001435	Dextromethorphans
Drug_1324	21.64310954	9.100693614	Otolaryngologic vasoconstrictors
Drug_2239	41.43109541	23.92968189	Expectorants, Others
Drug_2260	41.16607774	23.96555848	Gargles
Drug_2229	54.41696113	35.75699593	Antitussives, Others
Drug_2259	37.54416961	21.39440325	Bronchodilators, Others
Drug_1141	77.82685512	59.45945946	Anilines
Drug_2316	53.35689046	35.2068883	Probiotics
Drug_2329	86.04240283	69.97129873	Peptic ulcer agents, Others

ident_pct: proportion of individuals having a record of the code in the selected cluster.
others_pct: proportion of individuals having a record of the code in all other clusters.

Table is sorted in ascending order by the P-value (P-value < 0.05).
Rows in bold type indicate clinical characteristics of that cluster.

Supplementary Table 4C. Medical procedure codes for Cluster 4.

class	ident_pct	others_pct	Description
**Proc_J97**	**50.97173145**	**19.3613968**	**Nasal Medical procedure**
**Proc_J114**	**45.58303887**	**18.18942837**	**Nebulizer**
Proc_D273	68.99293286	36.78545802	Slit lamp microscopy (anterior segment)
Proc_D261	64.31095406	33.1260464	Refraction test
Proc_D263	66.60777385	37.58670175	Corrective vision test
Proc_D264	67.75618375	38.72279359	Precision tonometry
Proc_D265	52.47349823	27.18249223	Precision fundus examination (unilateral)
Proc_D255	65.37102473	38.28031571	Precision fundus examination (unilateral)
**Proc_J99**	**27.56183746**	**9.794307582**	**Indirect laryngoscopic laryngeal procedure**
**Proc_D12**	**86.83745583**	**61.65988998**	**Infectious disease immunological test**
**Proc_D419**	**62.36749117**	**38.79454676**	**Sample collection**
Proc_E2	94.5229682	74.94618512	Radiography
Proc_E1	94.61130742	75.08969146	X-ray diagnosis
Proc_E0	95.75971731	77.57713466	Fluoroscopic diagnosis
Proc_J98	18.46289753	6.541497249	Oral and pharyngeal procedures
Proc_J115	19.43462898	7.510165032	Ultrasonic nebulizer (per day)
Proc_J95	20.22968198	8.718010045	Ear treatment (including ear bath and ear cleaning)
Proc_D299	19.16961131	8.143984693	Laryngeal fiberscopy
Proc_D262	15.6360424	7.103563741	Adjustment test
Proc_F0	84.18727915	70.31810572	Dispensing fee

ident_pct: proportion of individuals having a record of the code in the selected cluster.
others_pct: proportion of individuals having a record of the code in all other clusters.

Table is sorted in ascending order by the P-value (P-value < 0.05).
Rows in bold type indicate clinical characteristics of that cluster.

Supplementary Table 5A. ICD-10 codes for Cluster 5.

class	ident_pct	others_pct	Description
**ICD10_E119**	**26.23906706**	**4.536326048**	**Non-insulin-dependent diabetes mellitus without complications**
ICD10_K590	51.89504373	19.05493207	Constipation
ICD10_T818	11.66180758	0.874187832	Other complications of procedures, not elsewhere classified
**ICD10_I509**	**29.83479106**	**8.694624926**	**Heart failure, unspecified**
**ICD10_M6259**	**8.649173955**	**0.578854105**	**Muscle wasting and atrophy, not elsewhere classified**
ICD10_I802	12.14771623	1.441228588	Phlebitis and thrombophlebitis of other deep vessels of lower extremities
ICD10_K210	59.2808552	28.38747785	Gastro-oesophageal reflux disease with oesophagitis
**ICD10_I500**	**18.27016521**	**3.768458358**	**Congestive heart failure**
ICD10_D509	23.71234208	7.395156527	Iron deficiency anaemia, unspecified
ICD10_T814	7.871720117	0.815121087	Infection following a procedure, not elsewhere classified
ICD10_Z518	5.344995141	0.25989368	Other specified medical care
ICD10_R579	7.677356657	0.838747785	Shock, unspecified
ICD10_Z966	7.191448008	0.744240992	Presence of orthopaedic joint implants
**ICD10_I10**	**83.57628766**	**57.30655641**	**Essential (primary) hypertension**
ICD10_R522	12.92517007	2.799763733	Other chronic pain
ICD10_K802	16.03498542	4.193738925	Calculus of gallbladder without cholecystitis
**ICD10_M8199**	**20.11661808**	**6.331955109**	**Osteoporosis, unspecified**
ICD10_E669	29.34888241	11.77790904	Obesity, unspecified
**ICD10_I209**	**22.44897959**	**8.068517425**	**Angina pectoris, unspecified**
**ICD10_M4806**	**20.69970845**	**7.0998228**	**Spinal stenosis, lumbar region**

ident_pct: proportion of individuals having a record of the code in the selected cluster.
others_pct: proportion of individuals having a record of the code in all other clusters.

Table is sorted in ascending order by the P-value (P-value < 0.05).
Rows in bold type indicate clinical characteristics of that cluster.

Supplementary Table 5B. Drug codes for Cluster 5.

class	ident_pct	others_pct	Description
**Drug_1119**	**72.303207**	**2.894270526**	**General anesthetics, Others**
**Drug_1229**	**65.59766764**	**2.480803308**	**Skeletal muscle relaxants, Others**
Drug_2160	30.6122449	2.114589486	Vasoconstrictors
Drug_2221	53.15840622	2.504430006	Ephedrines and Ephedra herbs
Drug_3319	97.86200194	28.96633196	Blood substitutes, Others
Drug_3334	63.94557823	5.245126994	Heparins
Drug_3929	59.76676385	5.93030124	Antidotes, Others
Drug_7219	61.51603499	10.59657413	X-ray contrast media, Others
Drug_3311	99.12536443	40.36621382	Physiological salines
Drug_2354	50.43731778	10.77377437	Phytochemical agents
Drug_2357	28.86297376	3.130537507	Glycerins
Drug_3259	23.32361516	2.06733609	Protein and amino acid preparations, Others
Drug_1214	95.62682216	45.52864737	Xylidines
Drug_2612	33.43051506	7.418783225	Iodates
Drug_2359	40.33041788	11.6479622	Purgatives, clysters, Others
Drug_3231	28.18270165	5.965741288	Glucoses
Drug_2119	9.912536443	0.496160662	Cardiotonics, Others
Drug_2344	50.14577259	18.97223863	Inorganic salts
Drug_6342	8.357628766	0.354400473	Blood components preparations
Drug_2139	27.11370262	6.745422327	Diuretics, Others

ident_pct: proportion of individuals having a record of the code in the selected cluster.
others_pct: proportion of individuals having a record of the code in all other clusters.

Table is sorted in ascending order by the P-value (P-value < 0.05).
Rows in bold type indicate clinical characteristics of that cluster.

Supplementary Table 5C. Medical procedure codes for Cluster 5.

class	ident_pct	others_pct	Description
Proc_A219	62.48785228	7.466036621	Recuperation environment addition (per day)
Proc_A234	88.53255588	13.19551093	Additional medical safety measures (first day of hospitalization)
**Proc_B6**	**32.55587949**	**2.150029533**	**Emergency lifesaving management fee**
Proc_B8	78.13411079	9.533372711	Drug management instruction fee
**Proc_D11**	**78.62001944**	**7.22976964**	**Immunohematological test**
**Proc_D13**	**96.11273081**	**31.93148258**	**Hepatitis virus-related tests**
**Proc_D200**	**61.71039845**	**7.714116952**	**Spirography and other tests**
**Proc_D205**	**60.64139942**	**7.725930301**	**Respiratory function test judgment fee**
Proc_D220	67.24975705	7.312463083	Respiratory heart rate monitoring, neonatal heart rate and respiration monitoring, cardioscope (Heartscope), cardiotachoscope
**Proc_D223**	**64.43148688**	**12.19137626**	**Percutaneous arterial oxygen saturation measurement (per day)**
**Proc_D225**	**25.94752187**	**0.921441229**	**Invasive arterial pressure measurement (including cost of catheter insertion and cost of fluoroscopy)**
**Proc_D6**	**97.57045675**	**23.65032487**	**Bleeding/coagulation test**
**Proc_H0**	**51.60349854**	**3.106910809**	**Cardiovascular disease rehabilitation fee**
**Proc_J2**	**45.48104956**	**1.169521559**	**Drainage method (per day)**
**Proc_J24**	**59.08649174**	**3.473124631**	**Oxygen inhalation (per day)**
**Proc_K931**	**19.5335277**	**0.153573538**	**Addition of ultrasonic coagulation and incision device, etc.**
**Proc_L8**	**69.77648202**	**2.020082693**	**Closed circulation general anesthesia with mask or endotracheal intubation**
**Proc_L9**	**39.65014577**	**1.358535145**	**Anesthesia management fee (I)**
**Proc_G4**	**89.79591837**	**31.34081512**	**Drip injection (per day)**
**Proc_A301**	**19.14480078**	**0.51978736**	**Specific intensive care unit management fee (per day)**

ident_pct: proportion of individuals having a record of the code in the selected cluster.
others_pct: proportion of individuals having a record of the code in all other clusters.

Table is sorted in ascending order by the P-value (P-value < 0.05).
Rows in bold type indicate clinical characteristics of that cluster.

Supplementary Table 6A. ICD-10 codes for Cluster 6.

class	ident_pct	others_pct	Description
ICD10_I10	17.97520661	64.94252874	Essential (primary) hypertension
ICD10_E785	9.194214876	49.56603331	Hyperlipidaemia, unspecified
ICD10_E14	5.681818182	42.28243021	Unspecified diabetes mellitus
ICD10_H522	12.19008264	44.06521229	Astigmatism
ICD10_M5456	9.090909091	38.30635703	Low back pain
ICD10_K210	8.26446281	34.40065681	Gastro-oesophageal reflux disease with oesophagitis
ICD10_E11	2.582644628	26.41332395	Non-insulin-dependent diabetes mellitus
ICD10_J304	24.79338843	52.4278677	Allergic rhinitis, unspecified
ICD10_E780	4.23553719	27.85596997	Pure hypercholesterolaemia
ICD10_M171	5.165289256	28.11400422	Other primary gonarthrosis
ICD10_J209	26.85950413	52.63898663	Acute bronchitis, unspecified
ICD10_E790	4.545454545	25.79169599	Hyperuricaemia without signs of inflammatory arthritis and tophaceous disease
ICD10_K760	4.958677686	25.9558996	Fatty (change of) liver, not elsewhere classified
ICD10_K295	10.74380165	33.36851982	Unspecified chronic gastritis
ICD10_J459	10.12396694	32.19563688	Asthma, unspecified
ICD10_K769	3.202479339	22.14403003	Liver disease, unspecified
ICD10_L309	16.6322314	38.85761201	Dermatitis, unspecified
ICD10_J029	17.66528926	40.01876613	Acute pharyngitis, unspecified
ICD10_K297	8.05785124	27.6683087	Gastritis, unspecified
ICD10_K590	6.404958678	24.45460943	Constipation

ident_pct: proportion of individuals having a record of the code in the selected cluster.
others_pct: proportion of individuals having a record of the code in all other clusters.

Table is sorted in ascending order by the P-value (P-value < 0.05).
Rows in bold type indicate clinical characteristics of that cluster.

Supplementary Table 6B. Drug codes for Cluster 6.

class	ident_pct	others_pct	Description
Drug_2329	27.7892562	76.89420596	Peptic ulcer agents, Others
Drug_2649	19.00826446	69.09453437	Analgesics, anti-itchings, astringents, anti-inflammatory agents, Others
Drug_1149	44.73140496	84.14262257	Antipyretics and analgesics, anti-inflammatory agents, Others
Drug_2149	10.53719008	50.15247478	Antihypertensives, Others
Drug_1214	16.42561983	54.87919306	Xylidines
Drug_3311	14.04958678	50.44569552	Physiological salines
Drug_3319	6.611570248	39.81937603	Blood substitutes, Others
Drug_2171	9.297520661	42.12995543	Coronary dilators
Drug_6132	37.60330579	69.31738213	Cephem antibioitics
Drug_2189	5.785123967	36.88716866	Hyperlipidemia agents, Others
Drug_1319	17.45867769	49.16725311	Ophthalmic agents, Others
Drug_4490	28.51239669	59.48862304	Allergic agents, Miscellaneous
Drug_3969	2.582644628	30.53014309	Antidiabetic agents, Others
Drug_1141	34.7107438	64.70795215	Anilines
Drug_5200	17.3553719	45.37884119	Traditional Chinese medicines
Drug_6241	20.55785124	47.23199625	Pyridonecarboxylic acids
Drug_2233	29.33884298	55.89960122	Cysteines
Drug_2399	20.45454545	46.80975839	Digestive organ agents, Miscellaneous
Drug_6149	20.55785124	46.0591133	Acting mainly on gram-positive bacteria and mycoplasma, Others
Drug_2454	10.22727273	31.97278912	Fluorinated adrenocorticotropic hormones

ident_pct: proportion of individuals having a record of the code in the selected cluster.
others_pct: proportion of individuals having a record of the code in all other clusters.

Table is sorted in ascending order by the P-value (P-value < 0.05).
Rows in bold type indicate clinical characteristics of that cluster.

Supplementary Table 6C. Medical procedure codes for Cluster 6.

class	Cluster 2	others_pct	Description
**Proc_A1**	**56.81818182**	**96.64555477**	**Follow-up examination**
**Proc_D0**	**19.52479339**	**82.01970443**	**Urinary Qualitative/semi-quantitative Examination (general substances)**
**Proc_D26**	**45.35123967**	**97.19680976**	**Specimen test judgment**
Proc_D400	21.90082645	92.17687075	Blood sampling (per day)
Proc_D5	17.87190083	90.17124091	Blood morphology/function test
Proc_D7	17.66528926	90.61693643	Blood chemistry tests
Proc_E0	19.4214876	86.59394792	Fluoroscopic diagnosis
Proc_E1	15.70247934	84.42411447	X-ray diagnosis
Proc_E2	15.49586777	84.29509735	Radiography
Proc_F400	62.60330579	96.39924935	Prescription recipe fee
Proc_D15	10.22727273	69.99765423	Immunoassay-Based Analysis of Plasma Protein
Proc_A0	69.00826446	96.2585034	Initial consultation
Proc_B0	20.76446281	67.90992259	Treatment for specific diseases
Proc_E203	10.84710744	57.65892564	Computerized tomography
Proc_D208	4.855371901	50.7154586	Electrocardiogram
Proc_D8	6.198347107	50.57471264	Endocrinological examination
Proc_D215	13.84297521	58.0811635	Ultrasound examination (including expenses required for recording)
Proc_B9	8.05785124	51.07905231	Charge for providing medical information(Ⅰ)
Proc_F0	38.2231405	75.80342482	Dispensing fee
Proc_E200	6.921487603	47.39619986	Computed tomography (CT) (per series)

ident_pct: proportion of individuals having a record of the code in the selected cluster.
others_pct: proportion of individuals having a record of the code in all other clusters.

Table is sorted in ascending order by the P-value (P-value < 0.05).
Rows in bold type indicate clinical characteristics of that cluster.

Supplementary Table 7A. ICD-10 codes for Cluster 7.

class	ident_pct	others_pct	Description
ICD10_I10	31.49425287	63.04499072	Essential (primary) hypertension
ICD10_H522	14.48275862	43.47170686	Astigmatism
ICD10_J209	27.47126437	52.28432282	Acute bronchitis, unspecified
ICD10_J304	28.3908046	51.75092764	Allergic rhinitis, unspecified
ICD10_E14	20.22988506	40.39888683	Unspecified diabetes mellitus
ICD10_E785	28.3908046	47.17068646	Hyperlipidaemia, unspecified
ICD10_H101	8.850574713	24.04916512	Acute atopic conjunctivitis
ICD10_K210	16.55172414	33.26762523	Gastro-oesophageal reflux disease with oesophagitis
ICD10_E780	11.72413793	26.83209647	Pure hypercholesterolaemia
ICD10_E11	10.57471264	25.33627087	Non-insulin-dependent diabetes mellitus
ICD10_J029	22.52873563	39.27411874	Acute pharyngitis, unspecified
ICD10_J069	24.94252874	41.23376623	Acute upper respiratory infection, unspecified
ICD10_E143	0.91954023	9.91419295	Unspecified diabetes mellitus with ophthalmic complications
ICD10_J459	17.24137931	31.22680891	Asthma, unspecified
ICD10_H269	1.609195402	10.57513915	Cataract, unspecified
ICD10_L309	23.44827586	37.9174397	Dermatitis, unspecified
ICD10_I509	2.528735632	11.8390538	Heart failure, unspecified
ICD10_K590	11.37931034	23.74768089	Constipation
ICD10_H109	5.172413793	15.45686456	Conjunctivitis, unspecified
ICD10_A099	15.97701149	28.8961039	Gastroenteritis and colitis of unspecified origin

ident_pct: proportion of individuals having a record of the code in the selected cluster.
others_pct: proportion of individuals having a record of the code in all other clusters.

Table is sorted in ascending order by the P-value (P-value < 0.05).
Rows in bold type indicate clinical characteristics of that cluster.

Supplementary Table 7B. Drug codes for Cluster 7.

class	ident_pct	others_pct	Description
Drug_2149	17.12643678	49.03756957	Antihypertensives, Others
Drug_1319	18.73563218	48.67810761	Ophthalmic agents, Others
Drug_2171	15.86206897	41.09461967	Coronary dilators
Drug_3311	23.67816092	49.06076067	Physiological salines
Drug_1141	40.22988506	63.81029685	Anilines
Drug_2189	13.33333333	35.77226345	Hyperlipidemia agents, Others
Drug_3969	9.540229885	29.5106679	Antidiabetic agents, Others
Drug_3319	17.47126437	38.34647495	Blood substitutes, Others
Drug_4490	37.12643678	58.26762523	Allergic agents, Miscellaneous
Drug_3327	20.8045977	40.7351577	Antiplasmins
Drug_1315	14.71264368	33.55751391	Ophthalmic cortisones
Drug_2399	26.55172414	45.89517625	Digestive organ agents, Miscellaneous
Drug_2233	35.74712644	54.9512987	Cysteines
Drug_2229	21.14942529	39.67996289	Antitussives, Others
Drug_6250	14.94252874	32.50231911	Antivirals
Drug_6241	28.16091954	46.16187384	Pyridonecarboxylic acids
Drug_5200	26.66666667	44.12105751	Traditional Chinese medicines
Drug_1214	34.94252874	52.5742115	Xylidines
Drug_3962	5.517241379	18.13543599	Biguanides
Drug_3334	1.83908046	12.59276438	Heparins

ident_pct: proportion of individuals having a record of the code in the selected cluster.
others_pct: proportion of individuals having a record of the code in all other clusters.

Table is sorted in ascending order by the P-value (P-value < 0.05).
Rows in bold type indicate clinical characteristics of that cluster.

Supplementary Table 7C. Medical procedure codes for Cluster 7.

class	ident_pct	others_pct	Description
Proc_D263	8.965517241	44.28339518	Corrective vision test
Proc_D264	10	45.43135436	Precision tonometry
Proc_D255	9.540229885	44.73562152	Precision fundus examination (unilateral)
Proc_F0	43.67816092	74.82606679	Dispensing fee
Proc_D261	7.126436782	39.84230056	Refraction test
Proc_D12	36.66666667	67.48608534	Infectious disease immunological test
Proc_D265	4.137931034	32.82699443	Precision fundus examination (unilateral)
Proc_D273	13.67816092	43.34415584	Slit lamp microscopy (anterior segment)
**Proc_B0**	**36.89655172**	**65.74675325**	**Treatment for specific diseases**
**Proc_B11**	**36.43678161**	**65.24814471**	**Provision of medical information**
Proc_D208	19.1954023	48.74768089	Electrocardiogram
Proc_F100	42.29885057	69.17903525	Prescription fee
Proc_D256	4.482758621	26.34508349	Fundus camera photography
Proc_D0	56.43678161	77.58580705	Urinary Qualitative/semi-quantitative Examination (general substances)
Proc_D257	3.793103448	23.85204082	Slit-lamp microscopy (anterior and posterior segments)
Proc_G4	17.01149425	39.76113173	Drip injection (per day)
Proc_D15	43.67816092	65.94387755	Immunoassay-Based Analysis of Plasma Protein
Proc_B9	25.74712644	48.80565863	Charge for providing medical information(Ⅰ)
Proc_D419	21.26436782	43.65723562	Sample collection
Proc_D5	67.24137931	84.36920223	Blood morphology/function test

ident_pct: proportion of individuals having a record of the code in the selected cluster.
others_pct: proportion of individuals having a record of the code in all other clusters.

Table is sorted in ascending order by the P-value (P-value < 0.05).
Rows in bold type indicate clinical characteristics of that cluster.

Supplementary Table 8. ICD-10, drug, and medical procedure codes indicating retinopathy in Cluster 2.

class	ident_pct	others_pct	Description
**ICD10_E143**	**29.69145169**	**3.671677531**	**Unspecified diabetes mellitus with ophthalmic complications**
**Proc_K282**	**9.863429439**	**0.944525742**	**Reconstructive lens surgery**
**ICD10_H353**	**10.11633789**	**1.928961022**	**Degeneration of macula and posterior pole**
**ICD10_H350**	**7.587253414**	**1.23719569**	**Background retinopathy and retinal vascular changes**
ICD10_H438	8.447142135	2.128508714	Other disorders of vitreous body
ICD10_H358	3.591299949	0.425701743	Other specified retinal disorders
Proc_K276	3.490136571	0.478914461	Retinal photocoagulation
ICD10_H348	2.984319676	0.319276307	Other retinal vascular occlusions
Proc_K280	1.820940819	0.039909538	Vitrectomy
ICD10_H431	1.871522509	0.172941333	Vitreous haemorrhage
ICD10_H356	2.124430956	0.266063589	Retinal haemorrhage
ICD10_H354	3.287809813	0.74497805	Peripheral retinal degeneration
ICD10_E113	1.77035913	0.212850871	Type 2 diabetes mellitus with ophthalmic complications
Proc_D258	1.365705615	0.186244512	Electroretinogram (ERG)
Proc_G16	1.416287304	0.226154051	Intravitreal injection
ICD10_H333	1.972685888	0.465611281	Retinal breaks without detachment
ICD10_H330	1.213960546	0.226154051	Retinal detachment with retinal break

ident_pct: proportion of individuals having a record of the code in the selected cluster.
others_pct: proportion of individuals having a record of the code in all other clusters.

Table is sorted in ascending order by the P-value (P-value < 0.05).
Rows in bold type indicate clinical characteristics of that cluster.

Supplementary Table 9A. ICD-10 codes indicating ocular diseases in Clusters 3 and 4.

class	Cluster 3	Cluster 4	Description
**ICD10_H522**	**19.6969697**	**65.45936396**	**Astigmatism**
**ICD10_H109**	**7.641633729**	**28.44522968**	**Conjunctivitis, unspecified**
**ICD10_H524**	**2.437417655**	**13.33922261**	**Presbyopia**
**ICD10_H041**	**6.060606061**	**18.10954064**	**Other disorders of lacrimal gland**
**ICD10_H101**	**23.91304348**	**40.28268551**	**Acute atopic conjunctivitis**
**ICD10_H269**	**1.646903821**	**8.303886926**	**Cataract, unspecified**
**ICD10_H160**	**1.581027668**	**7.862190813**	**Corneal ulcer**
**ICD10_H161**	**3.293807642**	**10.86572438**	**Other superficial keratitis without conjunctivitis**
**ICD10_H400**	**2.305665349**	**8.480565371**	**Glaucoma suspect**
**ICD10_H531**	**3.4914361**	**9.45229682**	**Subjective visual disturbances**
ICD10_H521	6.258234519	13.51590106	Myopia
ICD10_H438	1.185770751	5.300353357	Other disorders of vitreous body
ICD10_H010	5.204216074	11.57243816	Blepharitis
ICD10_H409	1.515151515	5.74204947	Glaucoma, unspecified
ICD10_H168	2.635046113	7.508833922	Other keratitis
ICD10_H001	1.712779974	5.830388693	Chalazion
ICD10_H259	0.197628458	2.650176678	Senile cataract, unspecified

Cluster 3: proportion of individuals having a record of the code in Cluster 3.
Cluster 4: proportion of individuals having a record of the code in Cluster 4.

Table is sorted in ascending order by the P-value (P-value < 0.05).
Rows in bold type indicate clinical characteristics of that cluster.

Supplementary Table 9B. Drug codes indicating ocular diseases in Clusters 3 and 4.

class	Cluster 3	Cluster 4	Description
**Drug_1319**	**36.29776021**	**71.81978799**	**Ophthalmic agents, Others**
**Drug_1315**	**34.65085639**	**62.80918728**	**Ophthalmic cortisones**
Drug_1317	0.724637681	3.356890459	Ophthalmic antibiotics

Cluster 3: proportion of individuals having a record of the code in Cluster 3.
Cluster 4: proportion of individuals having a record of the code in Cluster 4.

Rows in bold type indicate clinical characteristics of that cluster.
Table is sorted in ascending order by the P-value (P-value < 0.05).

Supplementary Table 9C. Medical procedure codes indicating ocular diseases in Clusters 3 and 4.

class	Cluster 3	Cluster 4	Description
**Proc_D261**	**12.77997365**	**64.31095406**	**Refraction examination**
**Proc_D263**	**14.82213439**	**66.60777385**	**Orthoptic Examination**
**Proc_D264**	**17.12779974**	**67.75618375**	**Precision tonometry**
**Proc_D255**	**16.46903821**	**65.37102473**	**Precision fundoscopy（One side）**
**Proc_D265**	**8.893280632**	**52.47349823**	**Precision Intraocular Pressure Measurement**
**Proc_D273**	**23.91304348**	**68.99293286**	**Slit-lamp microscopy (anterior segment)**
**Proc_D256**	**6.916996047**	**29.06360424**	**Fundus photography**
**Proc_D257**	**4.808959157**	**24.204947**	**Slit-lamp microscopy (anterior and posterior eye)**
**Proc_D262**	**2.503293808**	**15.6360424**	**Visual acuity test**
Proc_D260	2.371541502	11.13074205	Quantitative perimetry (One side)

Cluster 3: proportion of individuals having a record of the code in Cluster 3.
Cluster 4: proportion of individuals having a record of the code in Cluster 4.

Rows in bold type indicate clinical characteristics of that cluster.
Table is sorted in ascending order by the P-value (P-value < 0.05).

Supplementary Figure 1 : Flowchart of the study.

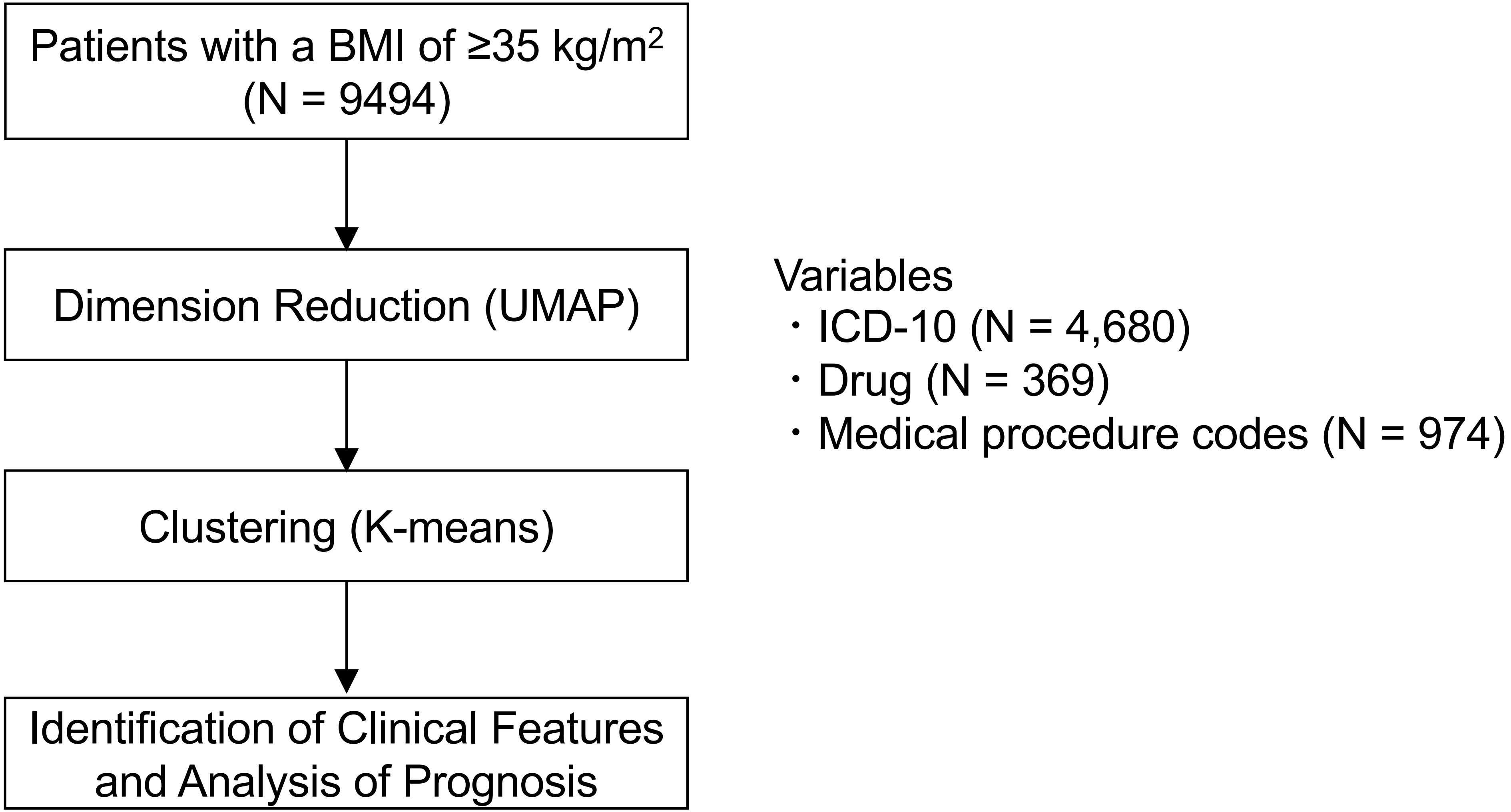

Supplementary Figure 2 : Elbow-Plot for K-means clustering

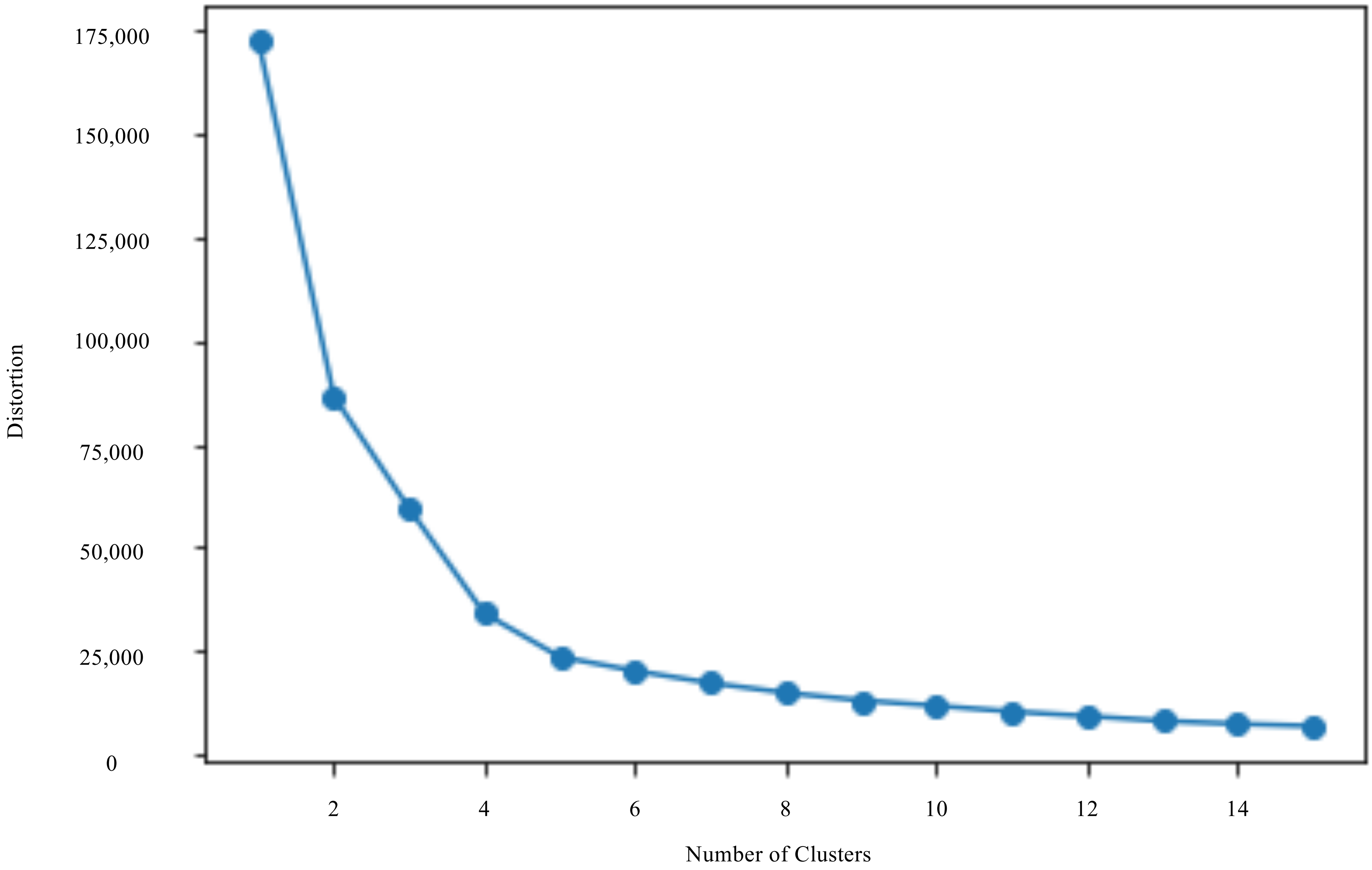

Supplementary Figure 3 :
Association between BMI and the proportion of the individuals with MHO.

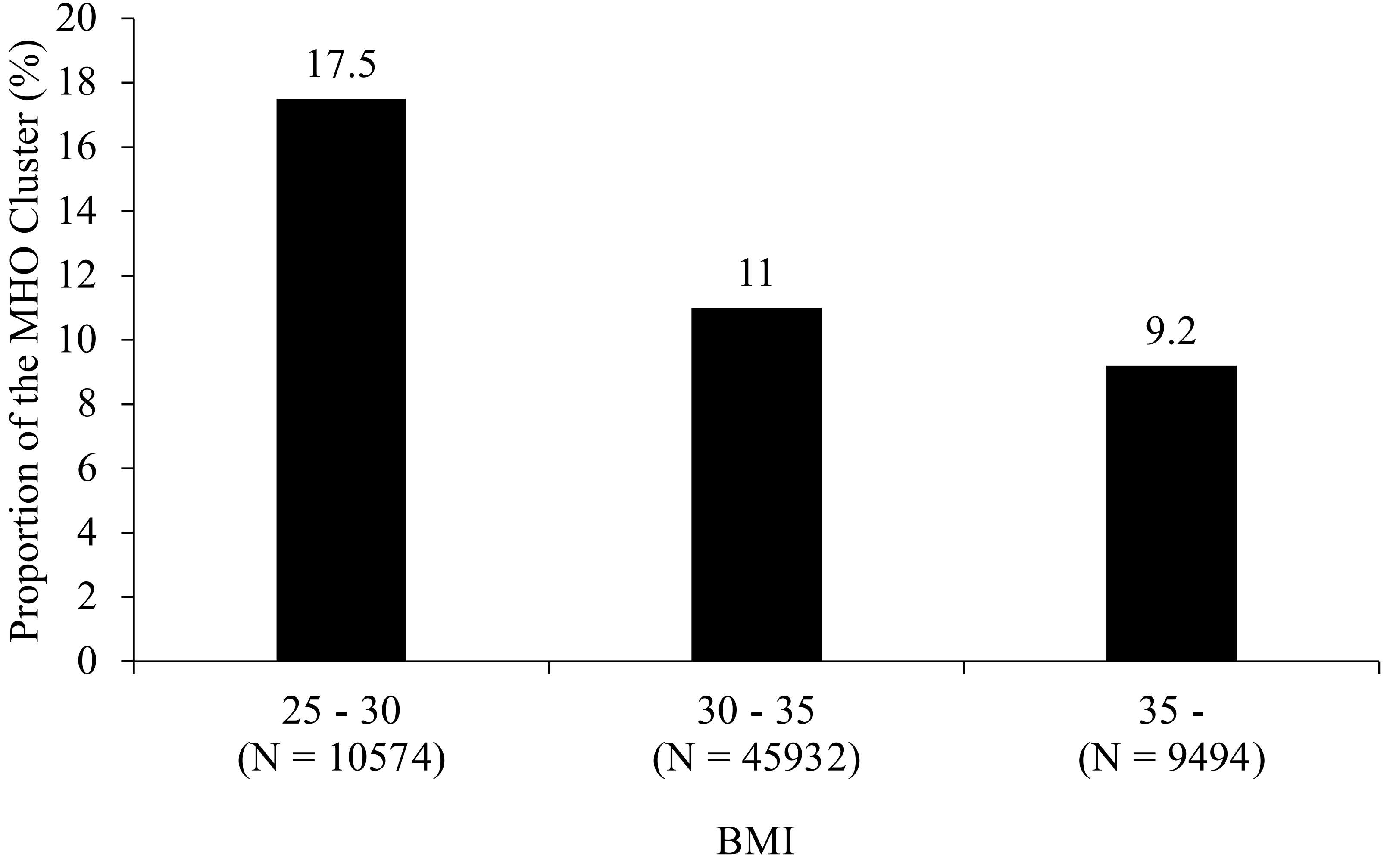

Supplementary Figure 4 : Health care fee of each cluster.

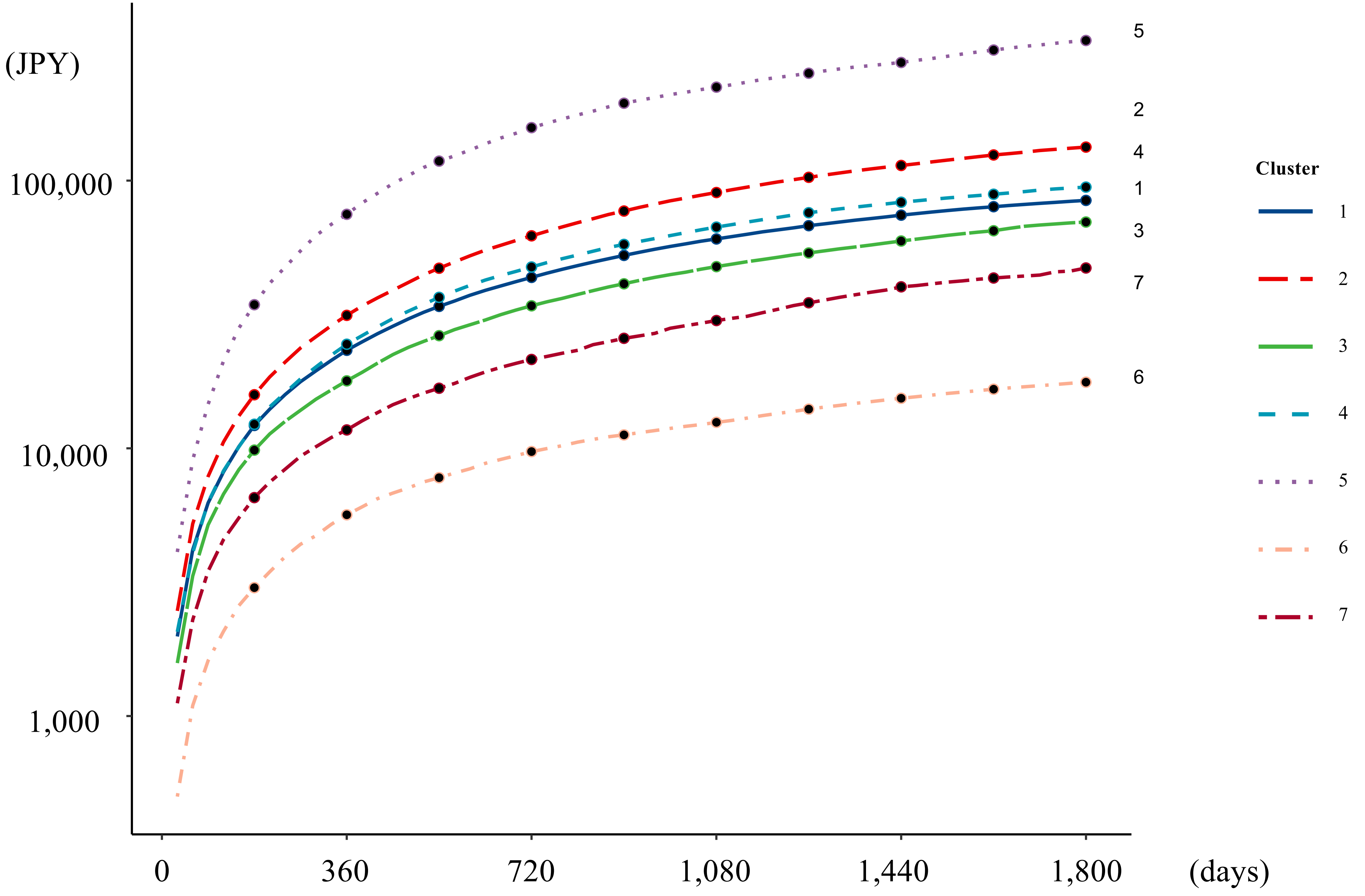

Supplementary Figure 5 : UMAP plots of all individuals with BMI 30 - 35.

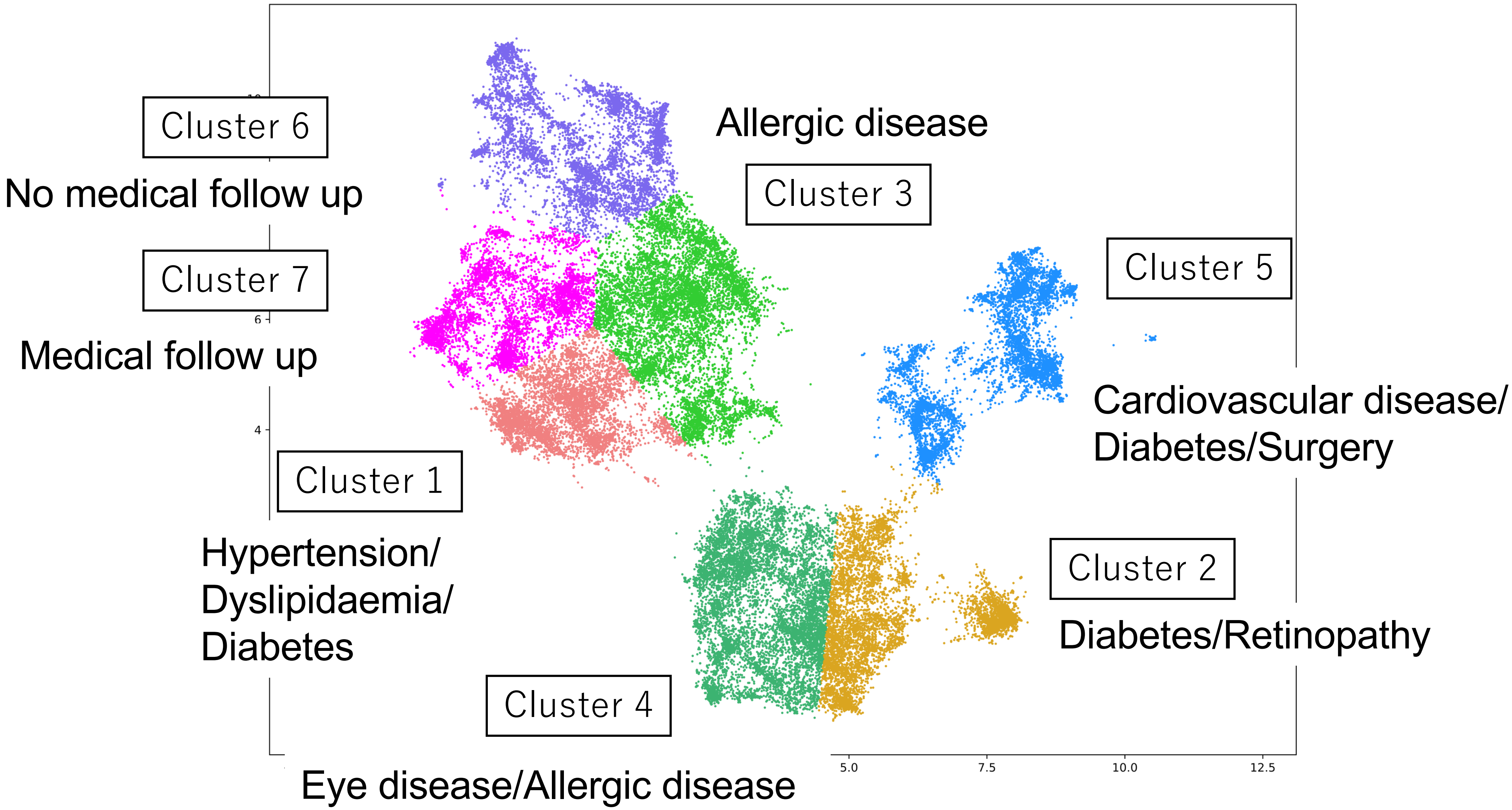

Supplementary Data 1: Python Code for UMAP Reduction and K-means Clustering

In [ ]:

```
import numpy as np
import pandas as pd
from sklearn.datasets import load_digits
import umap
import matplotlib.pyplot as plt
from kneed import KneeLocator
from sklearn.datasets import make_blobs
from sklearn.cluster import KMeans
from sklearn.metrics import silhouette_score
from sklearn.preprocessing import StandardScaler
```

In [ ]:

```
a = pd.read_csv('/path/to/binary_variables_BMI35.csv', delimiter=',',encoding='cp932')
```

In [ ]:

```
#umap
reducer = umap.UMAP(random_state=42,
                    min_dist=0.01,
                    n_neighbors=3,
                    n_components = 2,
                    unique=True
                   ).fit(a)
embedding = reducer.transform(a)
```

In [ ]:

```
#k-means Clustering
kmeans = KMeans(
    init="random",
    n_clusters=7,
    n_init=10,
    max_iter=300,
    random_state=42
)
```

In [ ]:

```
kmeans.fit(embedding)
```

In [ ]:

```
#Coloring
labels = [i for i in kmeans.labels_]

color_codes = ["goldenrod","limegreen","magenta","dodgerblue","mediumslateblue","lightcoral","mediumseagreen"]

colors = [color_codes[x] for x in labels]
import collections


l = []
for i in labels:
    l.append([i])
```

In [ ]:

```
#Plot
fig = plt.figure(figsize=(12.0, 8.0))
plt.scatter(embedding[:, 0], embedding[:, 1], c = colors,s=1)
plt.gca().set_aspect('equal', 'datalim')
```

Supplementary Data 2: Hierarchical Clustering

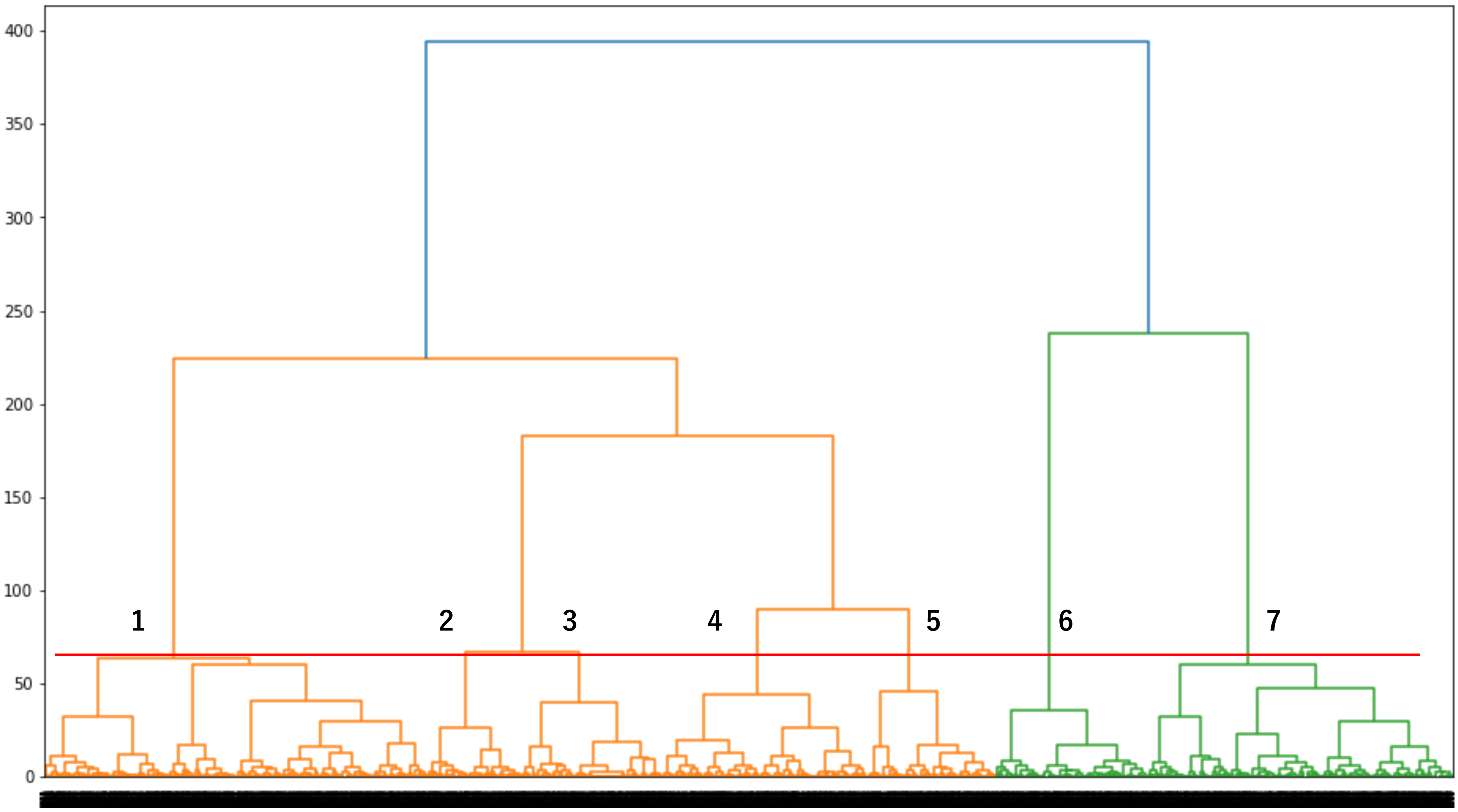

Supplement: Supplementary file 1 — Additional file 1: Supplementary Table 1A. ICD-10 codes for Cluster 1. Supplementary Table 1B. Drug codes for Cluster 1. Supplementary Table 1C. Medical procedure codes for Cluster 1. Supplementary Table 2A. ICD-10 codes for Cluster 2. Supplementary Table 2B. Drug codes for Cluster 2. Supplementary Table 2C. Medical procedure codes for Cluster 2. Supplementary Table 3A. ICD-10 codes for Cluster 3. Supplementary Table 3B. Drug codes for Cluster 3. Supplementary Table 3C. Medical procedure codes for Cluster 3. Supplementary Table 4A. ICD-10 codes for Cluster 4. Supplementary Table 4B. Drug codes for Cluster 4. Supplementary Table 4C. Medical procedure codes for Cluster 4. Supplementary Table 5A. ICD-10 codes for Cluster 5. Supplementary Table 5B. Drug codes for Cluster 5. Supplementary Table 5C. Medical procedure codes for Cluster 5. Supplementary Table 6A. ICD-10 codes for Cluster 6. Supplementary Table 6B. Drug codes for Cluster 6. Supplementary Table 6C. Medical procedure codes for Cluster 6. Supplementary Table 7A. ICD-10 codes for Cluster 7. Supplementary Table 7B. Drug codes for Cluster 7. Supplementary Table 7C. Medical procedure codes for Cluster 7. Supplementary Table 8. ICD-10, drug, and medical procedure codes indicating retinopathy in Cluster 2. Supplementary Table 9A. ICD-10 codes indicating ocular diseases in Clusters 3 and 4. Supplementary Table 9B. Drug codes indicating ocular diseases in Clusters 3 and 4. Supplementary Table 9C. Medical procedure codes indicating ocular diseases in Clusters 3 and 4. Supplementary Fig. 1. Flowchart of the study. Supplementary Fig. 2. Elbow-Plot for K-means clustering Supplementary Fig. 3. Association between BMI and the proportion of the individuals with MHO. Supplementary Fig. 4. Health care fee of each cluster. Supplementary Fig. 5. UMAP plots of all individuals with BMI 30-35. Supplementary Data 1. Python Code for UMAP Reduction and K-means Clustering. Supplementary Data 2. Hierarchical Clustering. [file 12889_2024_17648_MOESM1_ESM.pdf]
